# The NRF2/ID2 Axis in Vascular Smooth Muscle Cells: Novel Insights into the Interplay between Vascular Calcification and Aging

**DOI:** 10.14336/AD.2024.0075

**Published:** 2024-05-20

**Authors:** Mulin Xu, Xiuxian Wei, Jinli Wang, Yi Li, Yi Huang, Anying Cheng, Fan He, Le Zhang, Cuntai Zhang, Yu Liu

**Affiliations:** ^1^Department of Geriatrics, Tongji Hospital of Tongji Medical College, Huazhong University of Science and Technology, Wuhan, China.; ^2^Key Laboratory of Vascular Aging, Ministry of Education, Tongji Hospital of Tongji Medical College, Huazhong University of Science and Technology, Wuhan, China.; ^3^Department of General Medicine, Tongji Hospital of Tongji Medical College, Huazhong University of Science and Technology, Wuhan, China.; ^4^Department of Nephrology, Tongji Hospital of Tongji Medical College, Huazhong University of Science and Technology, 1095 Jiefang Avenue, Wuhan, China.

**Keywords:** Vascular Calcification, VSMC, Aging, NRF2, ID2

## Abstract

Vascular calcification (VC) increases with age and markedly exacerbates the risk of cardiovascular morbidity and mortality. However, effective pharmaceutical interventions are lacking and the molecular mechanisms linking aging to VC remain elusive. This study explored the role of nuclear factor erythroid 2-related factor 2 (NRF2) in age-associated VC, specifically focusing on vascular smooth muscle cell (VSMC) senescence. Using a chronologically aging mouse model, we noted a significant decline in the expression of NRF2 in the aged mice aortas, coinciding with increased VC. Administering NRF2 activators effectively reduced calcification. By establishing adenine-and vitamin D-induced VC models in VSMC-specific *Nrf2* knockout (*Nrf2^SMCKO^*) mice, there was an increase in VC with increased VSMC senescence. Aortic rings and primary VSMCs from *Nrf2^SMCKO^* mice also showed increased VC under high-phosphate conditions. Furthermore, *Nrf2* overexpression inhibited VSMC calcification with decreased VSMC senescence and an osteogenic phenotype, whereas *Nrf2* silencing aggravated calcification. Transcriptome RNA-seq analysis of the aortas from *Nrf2^SMCKO^* and control mice revealed that inhibitor of DNA binding 2 *(Id2*) is a core downstream gene of NRF2. *Id2* overexpression alleviated *NRF2* knockdown-induced VC and VSMC senescence, while silencing *Id2* negated the protective effects of NRF2. Moreover, results of a dual luciferase reporter assay indicated that NRF2 promotes the transcriptional activity of the *Id2* gene promoter region. This study emphasizes the critical role of age-related NRF2 dysfunction in the nexus between VSMC senescence and VC. The NRF2-ID2 axis in VSMCs has been proposed as a promising therapeutic target for reducing VC and mitigating age-related cardiovascular diseases.

## INTRODUCTION

Vascular calcification (VC) in the tunica media of a blood vessel wall, marked by ectopic deposition of calcium phosphate crystals, is common in elderly individuals and those with chronic kidney disease (CKD) or diabetes mellitus (DM) [1]. Unlike atherosclerosis, this systemic vascular disorder increases arterial stiffness [2], leading to diastolic heart failure [3] and reduced blood flow to critical organs, such as the brain, kidneys, liver, and limbs. Previously considered a passive degeneration, VC is now understood to be an active process, similar to bone formation [4]. Vascular smooth muscle cells (VSMCs) play a vital role in the process. The progression of VC hinges on the balance between pro-calcifying and anti-calcifying factors. This imbalance can induce senescence and osteogenic differentiation of VSMCs [5].

Recent studies have highlighted that aging affects the frequency and intensity of calcium deposition in blood vessels [6, 7]. However, the exact mechanisms are not fully understood. The accumulation of senescent VSMCs in aged blood vessels leads to biological dysfunction and emergence of age-related cardiovascular diseases. An increased senescent cell burden has also been confirmed in calcified aortas from 5/6-nephrectomized rats and in the epigastric arteries of patients with CKD [8]. Senescent VSMCs often display an osteoblastic phenotype characterized by elevated levels of bone morphogenetic protein 2 (BMP2), RUNX family transcription factor 2 (RUNX2), alkaline phosphatase (ALP), and type I collagen [9, 10]. The pro-calcification phenotype of senescent VSMCs may contribute to the pathophysiology of VC. A direct link between senescent VSMCs and age-associated VC as well as a thorough understanding of the regulatory mechanisms involved are still lacking.

Imbalances between the oxidative and antioxidant systems are a notable consequence of aging. Oxidative stress (OS) plays a pivotal role in age-associated VC [11]. Nuclear factor erythroid 2-related factor 2 (NRF2) is a transcription factor that plays a crucial role in the regulation of the expression of numerous antioxidant genes [12]. Age-related NRF2 dysfunction can increase production of reactive oxygen species (ROS) in the aortas of primates and rodents [13, 14]. Evidence suggests that activation of NRF2 by pharmacological agents and natural compounds can prevent VC in vitro and in vivo [15-20]. Molecules associated with NRF2, both upstream and downstream, have been shown to inhibit VC, including KEAP1, NQO1, HO-1, and P62 [20-22]. In vitro, silencing *Nrf2* or accelerating degradation of NRF2 promotes VSMC calcification, induced by a high phosphate level. In turn, overexpressing *Nrf2* or retarding degradation of NRF2 inhibits VSMC calcification [18, 21-23]. Notably, the NRF2 activator, Exendin-4, is effective in reducing angiotensin II-induced VSMC senescence [24]. However, the relationship between VSMC senescence, NRF2 dysfunction, and VC has not yet been fully established. These findings highlight the need for further studies on the intricate relationship between NRF2 and VSMC senescence in the context of VC.

In this study, we found that aging accelerated VC in a chronologically aging mouse model that was characterized by increased VSMC senescence and reduced apoptosis. A significant decrease in the expression of NRF2 was also observed in the aortas of aged mice. Administration of NRF2 activators effectively alleviated VC in aged mice. We established the VSMC-specific *Nrf2* knockout (*Nrf2^SMCKO^*) mice and built adenine-and vitamin D-induced VC models. *Nrf2^SMCKO^* mice showed increased medial VC and VSMC senescence compared with control (*Nrf2^WT^*) mice. Aortic rings and primary VSMCs from *Nrf2^SMCKO^* mice also showed increased VC under high-phosphate conditions. Furthermore, *Nrf2* silencing aggravated VSMC calcification and increased VSMC senescence, whereas *Nrf2* overexpression inhibited VSMC calcification and decreased VSMC senescence. Transcriptome RNA-seq analysis of the aortas from *Nrf2^SMCKO^* and control mice revealed that inhibitor of DNA binding 2 (*ID2*) is a core downstream target gene of NRF2. Overexpression of *Id2* alleviated NRF2 knockdown-induced VC and VSMC senescence while silencing *Id2* negated NRF2's protective effects against VC and VSMC senescence. Moreover, a dual-luciferase reporting assay indicated that NRF2 promotes transcription of the *Id2* gene promoter region. Our findings indicate that the NRF2-ID2 axis in VSMC senescence offers a promising avenue for understanding the association between aging and VC.

## MATERIALS AND METHODS

### Cell Culture

Primary mouse VSMCs were isolated as previously described [25]. Mice were euthanized with intraperitoneal sodium pentobarbital (150 mg/kg), and the thoracic aortas from 2-month-old males were harvested. The adventitia and intima were removed, and the aortic media were minced and digested in elastase. Passages three to eight of the primary mouse VSMCs were used. The MOVAS cell line was obtained from ATCC (Manassas, VA, USA). Primary mouse VSMCs were cultured in Dulbecco's Modified Eagle Medium/Nutrient Mixture F-12 (DMEM/F-12) (Thermo Fisher Scientific) and MOVAS cells were cultured in DMEM, both were supplemented with 10% fetal bovine serum (FBS), 100 U/ml penicillin and 100 mg/ml streptomycin, at 37°C in 5% CO_2_. VSMC calcification was induced in a calcifying medium (DMEM, 10% FBS, 2.6 mM inorganic phosphate) for seven days, with medium changes every two days.

### Animal Studies

Animal protocols were approved by the Institutional Animal Ethics Committee of Huazhong University of Science and Technology (IACUC Number: 3423) and followed National Institutes of Health (NIH) guidelines (8^th^ Edition, 2011). We used the Cre/LoxP strategy to generate Nrf2-VSMC-specific knockout (*Nrf2*^SMCKO^) mice (Fig. 2A). *Nrf2^flox/flox^* mice were obtained from the Pennington Biomedical Research Center/LSU System [26]. The Tagln-Cre mice (Cat. NO. NM-KI-200144) were purchased from the Shanghai Model Organisms Center, Inc. The F1 generation was backcrossed to obtain *Tagln-Cre^/+^; Nrf2^flox/+^* mice, which were further crossed to generate *Nrf2^SMCKO^* (*Tagln-Cre^/+^; Nrf2^flox/flox^*) and *Nrf2^WT^* (*Tagln-Cre^/-^; Nrf2^flox/flox^*) littermates. In the young group, C57BL/6J male mice aged 2-3 months were included, whereas in the old group, C57BL/6J male mice aged 18-20 months were included. The animals were maintained on a 12-h light/dark cycle and given free access to pellets and water.

### Vitamin D-mediated VC mouse model

*Nrf2^SMCKO^* and *Nrf2^WT^* littermates (aged 8 weeks) were randomly assigned to either the Vitamin D treatment or control groups. In the Vitamin D treatment group, the mice were subcutaneously injected with vitamin D3 (6 or 8 x 10^5^ IU/kg; C1357, Sigma-Aldrich, USA) daily for three days (n=5-8 per group). The Vitamin D3 solution (2.64 x 10^6^ IU) was prepared as previously described [27]. Briefly, Vitamin D3 (66 mg) in 200 μl of absolute ethanol was mixed with 1.4 ml of cremophor (S6828, Selleck, USA) and then with 18.4 ml of sterilized water containing 750 mg of dextrose. In the control group, the mice were subcutaneously injected with sterilized water containing dextrose (18.4 ml of sterilized water containing 750 mg of dextrose).

### Aortic Ring Organ Calcification

Thoracic arteries from eight-week-old *Nrf2^SMCKO^* and *Nrf2^WT^* male mice, and young and old C57BL/6J male mice were dissected and cut into 2-3 mm rings after removing the adventitia and intima. These were randomly incubated in either high-phosphate (3.8 mM inorganic phosphate) or regular DMEM at 37ºC in 5% CO_2_ for seven days, with medium changes every two days. Calcium deposition was assessed after seven days.

### Chronic Renal Failure Model

The adenine diet-induced Chronic Renal Failure (CRF) mouse model was established following an eight-week program as described previously [28]. Eight-week-old male mice were used in the present study. Adenine is mixed with a casein-based diet to conceal its taste and smell. The ingredients in the diet were maize starch (39.3%), casein (20.0%), maltodextrin (14.0%), sucrose (9.2%), maize/corn oil (5%), cellulose (5%), vitamin mix (1.0%), DL-methionine (0.3%), and choline bitartrate (0.2%). The total phosphate and calcium contents were 0.9% and 0.6 %, respectively. The control group was fed the same casein diet without the addition of adenine. *Nrf2^SMCKO^* and *Nrf2^WT^* mice were randomly divided into chow and adenine diet groups. All mice were fed a one-week chow diet followed by an eight-week distinct diet (chow or adenine), as shown in Figure 2C.

### Quantification of Calcium Content

The VSMCs and aortic rings were calcified in the calcifying medium. Thoracic aortas from CRF and Vitamin D-mediated VC mice were dissected, washed with phosphate-buffered saline (PBS) and incubated with 0.6 N HCl overnight at 4°C. The cell or tissue pellets were dissolved in 0.1 mol/L NaOH and 0.1% sodium dodecyl sulfate (SDS) for testing the concentration of protein. The calcium content in the supernatant was measured using the QuantiChrom Calcium Assay Kit (C004-2, Nanjing Jiancheng, China) and normalized to the overall protein concentration.

### Von Kossa staining and Alizarin Red S staining

For von Kossa staining, arterial sections were treated with 5% silver nitrate solution under ultraviolet light for 20-60 minutes; after which the unreacted sliver was removed by incubation with 5% sodium thiosulfate for 5 minutes. Nuclei were counterstained with hematoxylin. The calcified nodules were brown to black. For Alizarin Red S staining, the cells were washed three times with PBS, fixed with 10% formaldehyde, stained with 2% Alizarin Red S for 30 minutes, and washed with 0.2% acetic acid. Calcification was reddish/purple.

### Quantitative Real-Time PCR

Total RNA from the aortic tissues and VSMCs was reverse transcribed into cDNA. The HiScript RT Kit (R222, Vazyme, Nanjing, China) was used according to the instructions as previously described [29]. The primers used for the mice are listed in Supplement Table 1. Real-time PCR (RT-PCR) was conducted on a Light Cycler 480 II (Roche, Mannheim, Germany) using ChamQ SYBR qPCR Mix (Q711, Vazyme, Nanjing, China). The delta-delta Ct (2^-∆∆Ct^) method was used for analysis.

### Western Blot Analysis

VSMCs and arterial tissues were homogenized, and protein concentrations were quantified as previously described [29]. Proteins were separated by SDS-polyacrylamide gel electrophoresis (PAGE), transferred to polyvinylidene difluoride (PVDF) membranes and immunoblotted with specific primary antibodies: Nrf2 (GTX103322, 1:1000, GeneTex, USA), Nqo1 (11451-1-AP, 1:1000, Proteintech), Id2 (3431, 1:400, CST, USA), Caspase-3 (9662, 1:1000, CST, USA), Cleaved Caspase-3 (9664, 1:1000, CST, USA), GAPDH (10494-1-AP, 1:1000, Proteintech, USA), and β-Tubulin (ABL1030, 1:1000, Abbkine, China). Membranes were then incubated with horseradish peroxidase-conjugated secondary antibodies and visualized using an imaging system. Quantification was performed using the ImageJ software.

### Histological Analysis

For immunohistochemistry (IHC) staining, the slides were deparaffinized, and endogenous peroxidase activity was quenched with 3% (vol. /vol.) hydrogen peroxide for 10 minutes. After blocking nonspecific sites with 10% bovine serum in PBS, slides were incubated with primary and secondary antibodies, stained with diaminobenzidine, and counterstained with hematoxylin, as previously described [29]. The primary antibodies were: Nrf2 (GTX103322, 1:2000, GeneTex, USA), Id2 (3431, 1:40, CST, USA), Nqo1 (11451-1-AP, 1:500, Proteintech, USA), and Bmp2 (18933-1-AP, 1:100, Proteintech, USA). Isotype antibody controls, secondary antibody only controls, and tissue minus/plus controls were used to demonstrate the specificity of the antibodies and the specific interaction between primary antibody and its target antigen. Positive staining was analyzed using Image-Pro Plus software v. 6.0.

Immunofluorescence (IF) staining was performed as described previously [29]. Sections were blocked and incubated with primary and secondary antibodies, followed by DAPI staining, for 5 minutes, in the dark. The primary antibodies were: α-smooth muscle actin (α-SMA) (BM0002, 1:200, BOSTER, China), ID2 (3431, 1:70, CST, USA), p16 (ab54210, 1:100, Abcam, USA), and Nrf2 (GTX103322, 1:500, GeneTex, USA). Isotype antibody controls, secondary antibody only controls, and tissue minus/plus controls were used to demonstrate the specificity of the antibodies and the specific interaction between primary antibody and its target antigen. The fluorescence signal was monitored using a confocal laser-scanning microscope (Nikon C2+, Tokyo, Japan).

### Small interfering RNA and Lentivirus Transfection

VSMCs were transfected with small interfering RNA (siRNA) against Id2 or Nrf2 (RiboBio, Guangzhou, China) using Lipofectamine RNAiMAX Reagent (Invitrogen, Carlsbad, CA, USA). The target sequence of mouse siRNA-ID2 was 5’-GACCCAGTATTCGGTTA CT-3’. The target sequence of mouse siRNA-Nrf2 was 5'-GCATGATGGACTTGGAGTT-3'. Lentiviruses for full-length mouse Nrf2 (PubMed No. NM 010902) (LV-NRF2) (Shanghai Jikai Gene Chemical Technology Co., Ltd.) infected MOVAS cells according to the manufacturer's protocol. A lentivirus carrying green fluorescent protein (LV-GFP) was used as a negative control.

### TUNEL Assay

Aortic tissue slides were processed for in situ apoptosis detection using the TUNEL BrightGreen Apoptosis Detection Kit (A112; Vazyme, China). Cells were incubated with permeabilization solution containing Proteinase K (20 μg/ml) and then incubated with a rTdT reaction mixture containing fluorescein-12-dUTP at 3’-OH DNA TdT Enzyme (terminal transferase) following the manufacturer's instructions. Nuclei were stained with Hoechst and confocal microscopy was used for imaging. To quantify the extent of apoptosis, six sections with ten high-power fields (400 x magnification), from each, were averaged. The percentage of TUNEL-positive nuclei was calculated.

### Annexin V-FITC/PI Staining

Cell death was assessed using an Annexin V-FITC/PI Apoptosis Detection Kit (BD Pharmingen, USA). Following the treatments, the cells were collected and washed with cold PBS, and suspended in 195 μL of binding buffer. To stain the cells, 5 μL of Annexin V-FITC and 10 μL of PI (propidium iodide) were added to the suspension, and the mixture was incubated for 20 minutes, in the dark, at room temperature. After staining, flow cytometry analysis was immediately performed to determine the extent of apoptosis.

### γ-H2AX staining assay

The cells were fixed, permeabilized with Triton X-100, and blocked with 1% bovine serum albumin (BSA) in PBS for 2 hours. Cells were incubated with primary antibody (γ-H_2_AX, #9718, 1:200, CST, USA) and secondary antibody (AlexaFluor-555,1:1000, Invitrogen). γ-H_2_AX foci were assessed with red fluorescence.

### ROS Detection and Superoxide Dismutase (SOD) Activity Measurement

The intracellular ROS level was determined using dichloro-dihydro-fluorescein diacetate (DCFH-DA) (Beyotime, China). Briefly, after co-culture with t-BHP for 24 hours, the cells were harvested and incubated with DCFH-DA (10 μM), for 20 minutes, in the dark, at 37°C. The cells were washed three times with D/F12. Fluorescence signals were recorded using a flow cytometer (ACEA Biosciences, Inc., USA), and representative images were obtained using a fluorescence microscope. Dihydroethidium (DHE, HY-D0079, MedChemExpress, USA) was used to detect ROS production in Optimal Cutting Temperature (OCT)-embedded tissue sections as previously described^35^. SOD activity was measured using a SOD Activity Assay Kit (S0101, Beyotime, China).

**Figure 1.**
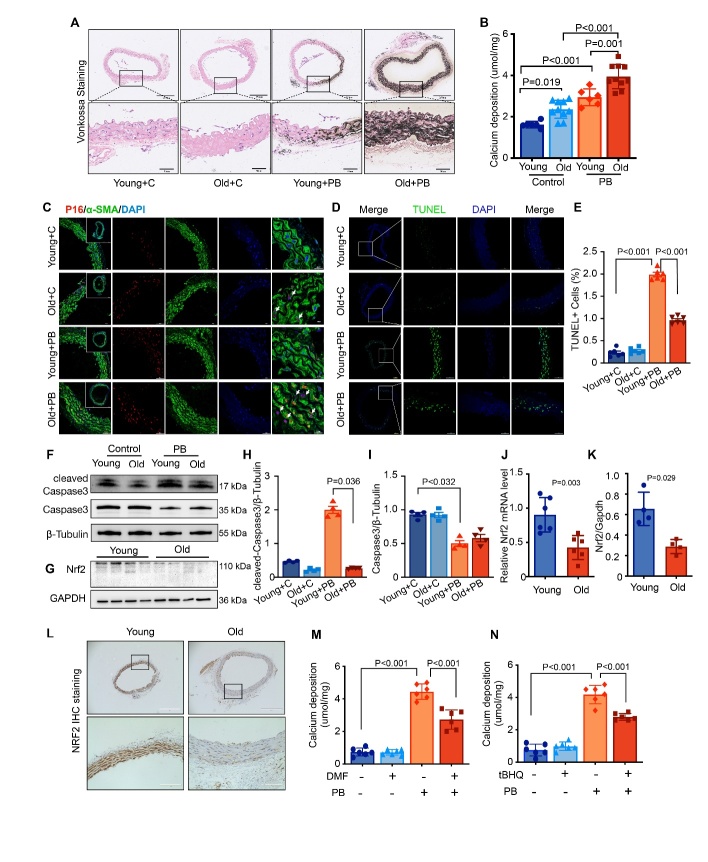
**Age Exacerbated VC and Reduced NRF2 Signal in Mice**. (**A**) Representative micrographs depict von Kossa staining in serial sections of aged and young mice aortas under normal or high-phosphate (High-Pi) conditions (Scale bars represent 250μm and 50μm). (**B**) Quantification of calcium deposition in the aortas of aged and young mice with or without High-Pi stimulation (n=6, 10, 6, and 10, respectively). (**C**) Immunofluorescence co-staining illustrates P16 (red) and α-SMA (α-smooth muscle actin; green) in serial sections of aged and young mice aortas under both conditions (Scale bars represent 100μm, 50μm and 20μm). Arrows indicate areas of intranuclear P16 upregulation. (**D-E**) Representative micrographs of TUNEL assay (Scale bars represent 100μm and 50μm) and percentage of TUNEL-positive cells in serial sections of aged and young mice aortas, with or without High-Pi stimulation (n=6 per group). (**F**) Western blot analysis of cleaved-Caspase3 and Caspase3 in aged and young mice aortas with or without High-Pi stimulation. (**G**) Western blot analysis of Nrf2 in aged and young mice aortas. (**H-I**) Quantification of western blot results of cleaved-Caspase3 and Caspase3 in aged and young mice aortas with or without High-Pi stimulation (relative to β-Tubulin, n=4 per group). (**J**) Quantitative real-time PCR results of Nrf2 in aged and young mice aortas (relative to β-actin, n=6 per group). (**K**) Quantification of Western blot results of Nrf2 in aged and young mice aortas (relative to GAPDH, n=4 per group). (**L**) Immunohistochemistry staining displaying Nrf2 in serial sections of aged and young mice aortas (Scale bars represent 400μm and 100μm). (**M-N**) Calcium deposition quantified in High-Pi induced aged mice aortas with or without NRF2 activator (DMF or tBHQ) intervention (n=6 per group). ‘C’ stands for ‘under normal condition’, ‘PB’ stands for ‘under high-phosphate condition’.

### RNA-Seq and Bioinformatics Analyses

RNA-seq libraries from 8-week-old *Nrf2^WT^* and *Nrf2^SMCKO^* mouse aortas were prepared using the VAHTS mRNA-seq v2 Library Prep Kit for Illumina, following the manufacturer’s recommendations, and index codes were added to attribute sequences to each sample. Qubit HS quantification and Agilent 2100 Bioanalyzer /Fragment Analyzer 5300 quality control yielded a final library size of approximately 350bp. RNA-Seq libraries were prepared and sequenced using the Illumina NovaSeq platform. Raw data (raw reads) in fastq format were first processed through primary quality control. HTSeq was used to count the number of reads that mapped to each gene. Differential expression analyses between the two conditions were performed using the DEGSeq R package (version 1.20.0). Differentially expressed genes were defined as those for which the adjusted P-value was below 0.05, and the absolute value of log^2^ (fold-change) was > 1. GO and KEGG enrichment analysis of differentially expressed gene sets were implemented in the GOseq R and KOBAS 3.0 packages, respectively. GO terms with an adjusted P-value below 0.05 were considered significantly enriched by differentially expressed genes. The Retrieval of Interacting Genes (STRING, http://string.embl.de/) database was used to construct a protein-protein interaction (PPI) network based on the differentially expressed gene sets, and the names of all interacting proteins and protein-coding genes were extracted from the network. A confidence score of ≥700 was set as the parameter for significance. After obtaining PPI relationships, a network diagram was constructed using Cytoscape 3.4.0. Raw data are available in the SRA database with accession number PRJNA1091835 (https://dataview.ncbi.nlm.nih.gov/object/PRJNA1091835). Differentially expressed genes (DEGs) identified in transcriptional profiles has been uploaded to Mendeley Data repository (https://data.mendeley.com/datasets). The human aortic transcriptomes from the NCBI Gene Expression Omnibus (GEO; www.ncbi.nlm.nih.gov/geo) database (GSE12644 and GSE83453) were normalized using Limma and computed for gene expression analysis.

### Dual Luciferase Reporter Assay

Wild-type and mutant reporter plasmids in the *Id2* promoter region (-1947 ~ +50bp) were synthesized and inserted into the PGL3-Basic vectors (pGL3-Basic-ID2-MT-luc and pGL3-Basic-ID2-MT-luc) (Genomeditech Co. Ltd.). HEK-293T cells were seeded in 24-well plates. Lipo2000 (1.5 μL), pRL-TK (25 ng), reporter plasmids (250 ng) and plasmids with target genes (250 ng) were transfected into 293T cells. A luminometer was used to detect the activities of Renilla and firefly luciferases. The results are presented as the ratio of firefly luciferase activity to Renilla luciferase activity. Empty vector-infected cells served as controls.

### Statistical Analysis

Data were analyzed using GraphPad Prism software (version 6.0). For normally distributed data, statistical significance was assessed using the Student’s t-test, Parametric two-way ANOVA and Tukey’s multiple comparisons test. For non-normally distributed data or a small number of data (n < 6), the nonparametric Mann Whitney test and Kruskal Wallis test were performed. Data are presented as the mean ± standard deviation (SD). All experiments were performed using at least four independent biological repeats (Supplement Table 2).

## RESULTS

### Age Exacerbated VC in Mice with Increased VSMC Senescence but Decreased Apoptosis

Advanced age is a well-established risk factor of VC. To explore age-related effects on VC, we used ex vivo aortic ring assays with high-phosphate (high-Pi) stimulation, which is a frequently used method for studying medial VC. In our study, C57BL/6J male mice aged 19-20 months (aged) and C57BL/6J male mice aged 2-3 months (young) were used to develop an aortic ring organ culture model. Figures 1A-B illustrates that calcification in the aortic ring was significantly induced by 3.8 mmol/L high-Pi stimulation over seven days, as evidenced by von Kossa staining and calcium deposition.

**Figure 2.**
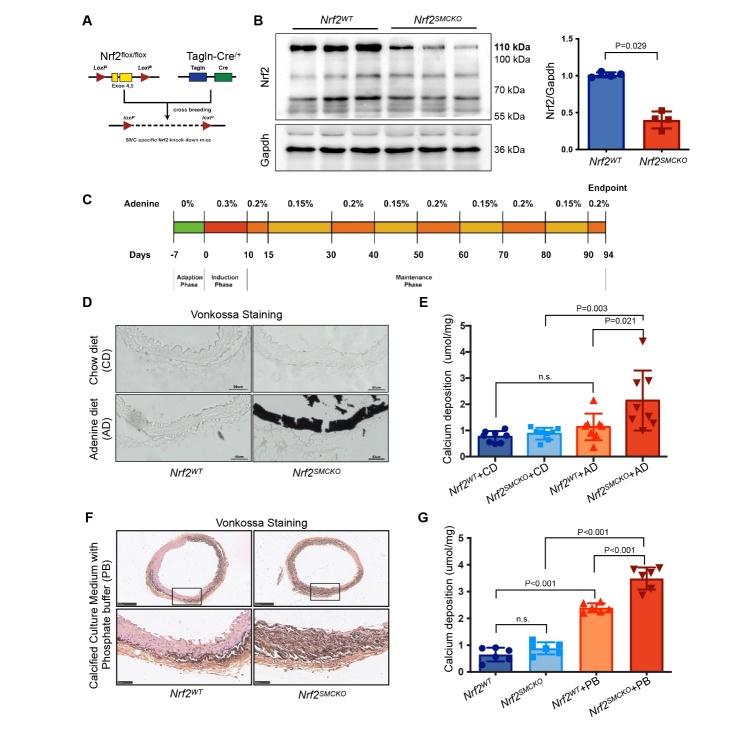
***Nrf2* Knockout in VSMC Aggravated Adenine Diet-induced VC**. (**A**) Generation strategy for VSMC-specific Nrf2 knockout (*Nrf2^SMCKO^*) mice: LoxP sites flank exon 4 and 5 of the Nrf2 gene. (**B**) Western blot analysis of Nrf2 expression in the aortas of *Nrf2^SMCKO^* (*Tagln-Cre^/+^; Nrf2^flox/flox^*) and *Nrf2^WT^* (*Tagln-Cre^/-^; Nrf2^flox/flox^*) littermates (relative to GAPDH, n=4 per group). (**C**) Schematic of the adenine diet protocol for inducing chronic renal failure in mice models. (**D**) Representative micrographs of von Kossa staining in serial sections of *Nrf2^SMCKO^* and *Nrf2^WT^* mice aortas with chow or adenine diet (Scale bars represent 50μm). (**E**) Quantitative analysis of calcium deposition in the aortas of *Nrf2^SMCKO^* and *Nrf2^WT^* mice with chow or adenine diet (n=8 per group). (**F**) Representative micrographs of von Kossa staining in serial sections of *Nrf2^SMCKO^* and *Nrf2^WT^* mice aortic rings cultured ex vivo with or without High-Pi treatment (Scale bars represent 250μm and 50μm). (**G**) Quantitative analysis of calcium deposition in *Nrf2^SMCKO^* and *Nrf2^WT^* mice aortic rings cultured ex vivo under High-Pi treatment (n=6 per group). ‘CD’ stands for ‘chow diet’, ‘AD’ stands for ’adenine diet’, and ‘PB’ stands for ‘under high-phosphate condition’.

Notably, aortic explants from aged mice showed significantly more calcification under high-Pi stimulation than those from young mice. Even in the absence of high-Pi stimulation, calcium deposition was marginally higher in the aortic rings of aged mice than in those of young mice, although von Kossa staining showed minimal differences between the two groups without high-Pi intervention. Correspondingly, OS levels were elevated in the aortic rings of aged mice with high-Pi stimulation, as depicted in Supplementary Figure 1A and B. IF staining demonstrated increased expression of P16 under high-Pi conditions, particularly in the aortic rings of aged mice. Moreover, the intranuclear expression of P16 significantly increased in the aortic rings of aged mice (Fig. 1C). While high-Pi conditions also triggered apoptosis in VSMCs, by TUNEL staining, the aortic rings of aged mice displayed significantly lower VSMC apoptosis under high-Pi conditions compared to younger mice, as shown in Figure 1D and E. Western blot and IHC staining of cleaved-caspases3 also showed a decreased expression level in the aortic rings of aged mice under high-Pi conditions (Fig. 1F, H, I and Supplementary Fig. 1E), suggesting that VSMCs in aged mouse aortas may be more resistant to apoptosis. Overall, our findings indicated that age exacerbates VC in mice, which is characterized by increased cell senescence and reduced apoptosis. The evidence indicates that VSMC senescence, rather than apoptosis, contributes to the onset of age-associated VC.

**Figure 3.**
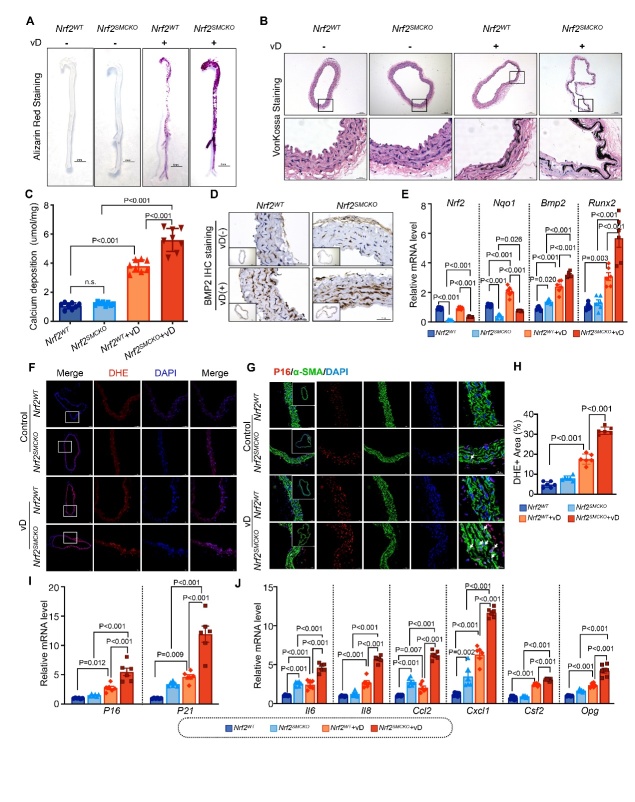
**NRF2 Deficiency in VSMC Contributes to Vitamin D-mediated VC and VSMC Senescence**. (**A**) Representative photographs of Alizarin Red S staining in the aortas of *Nrf2^SMCKO^* and *Nrf2^WT^* mice following injection with dextrose water or vitamin D, scale bars represent 5mm. (**B**) Representative photographs of von Kossa staining in serial sections of *Nrf2^SMCKO^* and *Nrf2^WT^* mice aortas post-injection with dextrose water or vitamin D, scale bars represent 200μm and 50μm. (**C**) Quantitative analysis of calcium deposition in the aortas of *Nrf2^SMCKO^* and *Nrf2^WT^* mice (n=8 per group). (**D**) Representative photographs of BMP2 immunohistochemistry staining in serial sections of *Nrf2^SMCKO^* and *Nrf2^WT^* mice aortas after dextrose water or vitamin D injection, scale bars represent 400μm and 50μm. (**E**) Quantitative real-time PCR for Nrf2, Nqo1, Bmp2, and Runx2 in the aortas of *Nrf2^SMCKO^* and *Nrf2^WT^* mice treated with dextrose water or vitamin D (relative to β-actin, n=6 per group). (**F**) Representative micrographs of DHE staining in serial sections of *Nrf2^SMCKO^* and *Nrf2^WT^* mice aortas injected with dextrose water or vitamin D, scale bars represent 100μm and 50μm. (**G**) Immunofluorescence staining for P16 (red) and α-SMA (green) in serial sections of *Nrf2^SMCKO^* and *Nrf2^WT^* mouse aortas injected with dextrose water or vitamin D, scale bars represent 100μm, 50μm and 20μm. Arrows indicate areas of intranuclear P16 upregulation. (**H**) Quantitative analysis of the percentage of DHE-positive areas in the aortas of *Nrf2^SMCKO^* and *Nrf2^WT^* mice treated with dextrose water or vitamin D (n=6 per group). (**I-J**) Quantitative real-time PCR for p16, p21, and SASP components (Il-6, Il-8, Ccl2, Cxcl1, Csf2, Opg) in the aortas of *Nrf2^SMCKO^* and *Nrf2^WT^* mice following dextrose water or vitamin D injection (relative to β-actin, n=6 per group). Scale bars represent 100μm, 50μm and 20μm. ‘vD’ stands for ‘vitamin D treatment’.

### Reduced NRF2 Signal Observed in the Aged Mouse Aortas

We further explored the impact of aging on *Nrf2* expression and activity in mouse aortas. Our results showed that both Nrf2 mRNA expression and NRF2 protein expression were substantially lower in the aortas of aged mice than in young mice, as demonstrated by RT-PCR, western blotting and IHC (Fig. 1G, J, K and L). Additionally, the mRNA level of *Nqo1*, an *Nrf2* target gene, was reduced in aged mice (Supplementary Fig. 1F), with a corresponding decrease in protein expression, as observed by western blotting and IHC staining (Supplementary Fig. 1G and H). Furthermore, IF assays indicated decreased NRF2 nuclear translocation in aged mouse aortas compared to young aortas under high-Pi conditions (Supplementary Fig. 1C and D), suggesting that aging diminishes NRF2 expression and its functional activity. In addition, the use of NRF2 activators, including DMF and tBHQ, effectively reduced calcification in aged aortic rings under high-Pi conditions, which was confirmed by observing calcium deposition (Fig. 1M and N). Therefore, diminished Nrf2 expression and activity in aged mouse aortas may be associated with increased susceptibility to VC.

### Nrf2 Knockout in VSMC Aggravated Adenine Diet-induced VC

To explore the relationship between NRF2 and VC, we generated *Nrf2*-VSMC-specific knockout mice (*Nrf2^SMCKO^, Tagln-Cre^/+^; Nrf2^flox/flox^*) by crossbreeding *Nrf2* conditional allele mice (*Nrf2^flox/flox^*) with Cre recombinase mice under the SM22α promoter (*Tagln-Cre^/+^*). Their littermates served as controls (*Nrf2^WT^, Tagln-Cre^/-^; Nrf2^flox/flox^*) (Fig. 2A and Supplementary Fig. 2A). We observed a significant reduction in Nrf2 expression in the aortas of *Nrf2^SMCKO^* mice compared to *Nrf2^WT^* mice (Fig. 2B). This reduction in Nrf2 expression was also noted in the uterus of *Nrf2^SMCKO^* mice, but no significant differences were found in other tissues (heart, liver, and skeletal muscle) when compared with *Nrf2^WT^* mice (Supplementary Fig. 2B and C). Given the high incidence of VC in CRF patients, we used an adenine-diet-induced CRF mouse model to explore the in vivo role of NRF2 in VC (Fig. 2C). The adenine diet induced significant VC in mice, as confirmed by von Kossa staining and calcium deposition. Notably, *Nrf2^SMCKO^* mice showed considerably more arterial medial calcification than *Nrf2^WT^* mice under CRF conditions (Fig. 2D and E). RT-PCR analysis revealed that the mRNA level of Bmp2 and Runx2 increased in *Nrf2^SMCKO^* mice aortas compared with *Nrf2^WT^* mice aortas under CRF conditions (Supplementary Fig. 2D and E). To confirm these findings, we performed additional ex vivo aortic ring assays. *Nrf2^WT^* mouse aortas exhibited mild VC under high-Pi conditions. In comparison, aortic rings from *Nrf2^SMCKO^* mice demonstrated a pronounced increase in calcium deposition and von Kossa staining under high-Pi conditions (Fig. 2F and G). Further, *Nrf2^SMCKO^* mice aortas showed more increased mRNA level of Bmp2 and Runx2 than *Nrf2^WT^* mice aortas under high-Pi conditions (Supplementary Fig. 2F and G).

### NRF2 Deficiency in VSMC Contributed to Vitamin D-mediated VC and VSMC Senescence

The Vitamin D-mediated VC mouse model is widely used to study medial VC owing to its rapid induction and high survival rate [27]. In our study, both *Nrf2^SMCKO^* and *Nrf2^WT^* mice were used to elucidate the protective role of NRF2 in medial VC. Nine days after Vitamin D injection, there was a marked increase in Alizarin Red S and von Kossa staining in the aortas of *Nrf2^WT^* and *Nrf2^SMCKO^* mice, with *Nrf2^SMCKO^* mice showing notably stronger staining than their littermates (Fig. 3A and B). This was paralleled by a similar trend in calcium deposition (Fig. 3C). Furthermore, IHC staining showed that Vitamin D treatment significantly induced BMP2 expression, which intensified in the absence of NRF2, leading to an increased BMP2 protein level (Fig. 3D). RT-PCR analysis revealed that *Nrf2* knockout increased the *Bmp2* and Runx2 mRNA level following Vitamin D treatment (Fig. 3E). Overall, the absence of NRF2 led to a marked increase in VC. Although vitamin D treatment slightly increased the mRNA level of Nrf2 in *Nrf2^SMCKO^* mice, the mRNA level of Nqo1 showed a more pronounced increase following Vitamin D treatment in both *Nrf2^SMCKO^* and control mice (Fig. 3E). DHE staining demonstrated that Vitamin D treatment increased OS in both *Nrf2^SMCKO^* and *Nrf2^WT^* mice and that *Nrf2* knockout further amplified this effect. However, no significant difference in the OS level was observed between *Nrf2^SMCKO^* and control mice before Vitamin D treatment (Fig. 3F and H). This was accompanied by increased VSMC apoptosis in *Nrf2^SMCKO^* mice, as determined by TUNEL staining (Supplementary Fig. 2H). IF staining revealed that Vitamin D treatment increased p16 expression, and NRF2 deficiency led to a further increase in the p16 protein level, especially the intranuclear expression of NRF2 (Fig. 3G). Furthermore, RT-PCR analysis showed that Vitamin D treatment increased the expression of cell senescence markers (p21 and p16) and SASP components (Il-6, Il-8, Cxcl1, Csf2, and Opg); these effects were more pronounced in the absence of NRF2 (Fig. 3I and J). Even without Vitamin D intervention, NRF2 deficiency alone elevated the level of SASP components (Il-6, Ccl2, and Cxcl1) (Fig. 3J). These results support the hypothesis that NRF2 plays an integral role in mitigating VC by regulating senescence and apoptosis in VSMCs.

**Figure 4.**
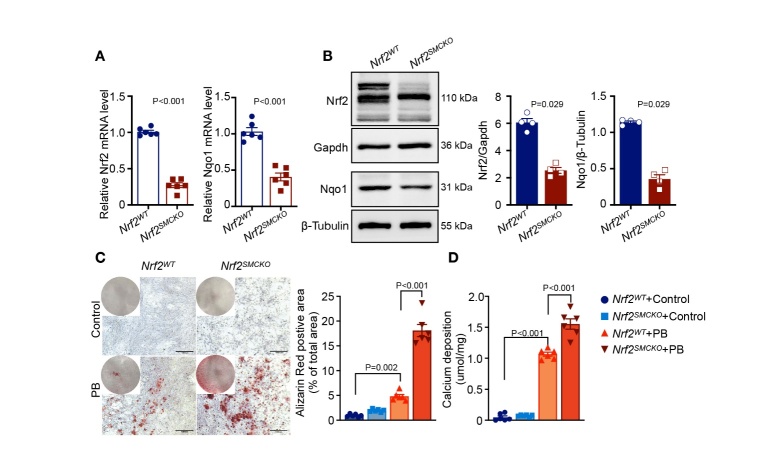
**NRF2 Deficiency Exacerbated VSMC Calcification**. (**A-B**) Quantitative real-time PCR (relative to β-actin, n=6 per group) and Western blot analyses (relative to GAPDH or β-tubulin, n=4 per group) assess Nrf2 and Nqo1 expression in mouse VSMCs derived from the aortas of *Nrf2^SMCKO^* and *Nrf2^WT^* mice. (**C**) Representative graphs of Alizarin Red S staining, both whole well and microscopic (scale bars represent 1000μm), alongside quantification of the percentage of Alizarin Red S-positive area in mouse VSMCs from the aortas of *Nrf2^SMCKO^* and *Nrf2^WT^* mice, with or without High-Pi stimulation (n=6 per group). (**D**) Quantitative analysis of calcium deposition in mouse VSMCs derived from the aortas of *Nrf2^SMCKO^* and *Nrf2^WT^* mice under both conditions (n=6 per group). ‘PB’ stands for ‘under high-phosphate condition’.

### NRF2 Deficiency Exacerbated VSMC Calcification

To further investigate the role of NRF2 in VSMC calcification, we used a high-Pi-induced VSMC calcification model and evaluated the effect of *Nrf2* knockdown on VC. We observed a pronounced reduction in NRF2 and expression of its downstream gene *Nqo*1 in primary VSMCs of *Nrf2^SMCKO^* mice compared to *Nrf2^WT^* mice (Fig. 4A and B). Although NRF2 ablation did not exacerbate calcium deposition in VSMCs under normal conditions; primary VSMCs from *Nrf2^SMCKO^* mice exhibited increased calcification susceptibility upon exposure to high Pi conditions (Fig. 4C and D). To further validate this effect, we employed small interfering RNA (siRNA) to knock down *Nrf2* and examined its direct role in driving VSMC calcification. The decrease in Nrf2 expression was substantial at both the mRNA and protein levels; a concurrent reduction in NQO1 expression was also confirmed (Supplementary Fig. 3A and B). Consistent with the observations in primary VSMCs of *Nrf2^SMCKO^* mice, *Nrf2* silencing alone did not lead to spontaneous calcification without a calcifying medium. However, under high-Pi conditions, *Nrf2* knockdown significantly intensified calcium deposition in VSMCs, in contrast to the control siRNA treatments (Supplementary Fig. 3C and D). Furthermore, with or without high-Pi intervention, the ROS level significantly increased, as evidenced by the DCFH-DA fluorescent probe (Supplementary Fig. 3H) and DHE staining (Supplementary Fig. 3E and G), whereas SOD activity decreased in *Nrf2* knockdown VSMCs (Supplementary Fig. 3I). Additionally, γ-H2AX IF staining showed that *Nrf2* knockdown aggravated DNA damage in high-Pi conditions (Supplementary Fig. 3E and F). These in vitro findings further supported our hypothesis that NRF2 serves as a key inhibitor of VSMC calcification.

**Figure 5.**
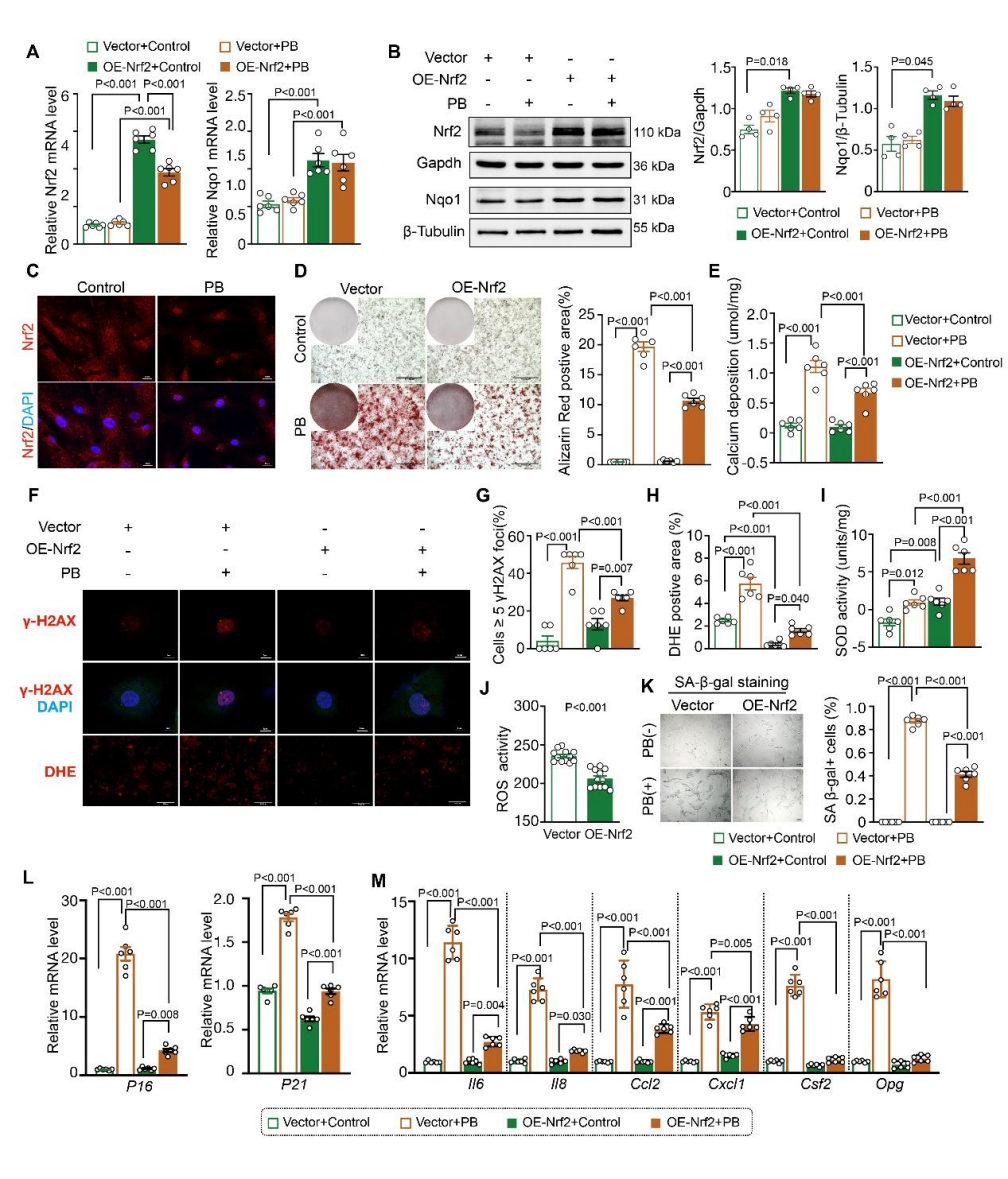
***Nrf2* Overexpression Ameliorated VSMC Calcification**. (**A**) Quantitative real-time PCR analysis of Nrf2 and Nqo1 in MOVAS cells treated with NRF2 lentivirus or control lentivirus, with or without High-Pi stimulation (relative to β-actin, n=6 per group). (**B**) Western blot analysis of NRF2 and NQO1 in MOVAS cells treated with NRF2 lentivirus or control lentivirus under both conditions (relative to GAPDH or β-tubulin, n=4 per group). (**C**) Representative micrographs of NRF2 immunofluorescence staining with or without High-Pi stimulation (scale bars represent 10μm). (**D**) Representative graphs of Alizarin Red S staining, both whole well and microscopic (scale bars represent 1000μm), and quantification of the percentage of Alizarin Red S-positive area in MOVAS cells treated with NRF2 lentivirus or control lentivirus under both conditions (n=6 per group). (**E**) Quantitative analysis of calcium deposition in MOVAS cells treated with NRF2 lentivirus or control lentivirus, with or without High-Pi stimulation (n=6 per group). (**F**) Representative micrographs of γ-H_2_AX (scale bars represent 5μm) and DHE staining (scale bars represent 500μm) in MOVAS cells treated with NRF2 lentivirus or control lentivirus under both conditions. (**G-H**) Quantitative analysis of cells with 5 or more γ-H_2_AX foci and DHE-positive areas in MOVAS cells treated with NRF2 lentivirus or control lentivirus, with or without High-Pi stimulation (n=6 per group). (**I**) Measurement of SOD (superoxide dismutase) activity in MOVAS cells treated with NRF2 lentivirus or control lentivirus under both conditions (n=6 per group). (**J**) Detection of intracellular ROS (reactive oxygen species) levels using DCFH-DA in MOVAS cells treated with NRF2 lentivirus or control lentivirus, with or without High-Pi stimulation (n=12 per group). (**K**) Representative micrographs of SA-β-gal staining (scale bars represent 100μm) and quantification of the percentage of SA-β-gal positive cells in MOVAS cells treated with NRF2 lentivirus or control lentivirus under High-Pi stimulation (n=6 per group). (**L-M**) Quantitative real-time PCR analysis of cell senescence markers (p16 and p21) and SASP components (Il-6, Il-8, Ccl2, Cxcl1, Csf2, Opg) in MOVAS cells treated with NRF2 lentivirus or control lentivirus under High-Pi stimulation (relative to β-actin, n=6 per group). ‘PB’ stands for ‘under high-phosphate condition’, ‘Vector’ stands for ‘OE-Control’.

### Nrf2 Overexpression Ameliorated VSMC Calcification

To determine whether *Nrf2* overexpression could mitigate VSMC calcification, we used lentivirus LV-NRF2 for NRF2 induction. Figure 5A and 5B illustrate that both the mRNA and protein levels of NRF2 and NQO1 significantly increased following LV-NRF2 infection compared to LV-Control infection without a high-Pi medium. High-Pi slightly reduced the mRNA level of Nrf2 following LV-NRF2 infection. However, there was no significant change in the NRF2 protein level. Similarly, Nqo1 expression remained largely unaffected by a high Pi level, with or without LV-NRF2 infection. Laser confocal microscopy demonstrated a significant enhancement in NRF2 nuclear translocation under high-Pi conditions (Fig. 5C and Supplementary Fig. 3J). Alizarin Red S staining, indicative of calcification, was notably intensified in control cells under high-Pi conditions; however, cells infected with LV-NRF2 showed a marked reduction in staining (Fig. 5D). This reduction was further corroborated by decreased calcium deposition, illustrating the protective role of NRF2s against VSMC calcification (Fig. 5E). Additionally, γ-H2AX IF staining showed that *Nrf2* overexpression mitigated DNA damage under high-Pi conditions (Fig. 5F and G). Concomitantly, the ROS level was significantly decreased, as evidenced by the DCFH-DA fluorescent probe (Fig. 5J) and DHE staining (Fig. 5F and H), whereas SOD activity increased in LV-NRF2 infected VSMCs (Fig. 5I), with or without high-Pi intervention. Notably, a high Pi level increased VSMC senescence, as indicated by a rise in SA-β-gal positive cells, an effect that was mitigated by *Nrf2* overexpression (Fig. 5K). Furthermore, RT-PCR analysis showed that a high Pi level induced an increase in cell senescence markers (p16 and p21) and SASP components (Il-6, Il-8, Ccl2, Cxcl1, Csf2, and Opg). Remarkably, *Nrf2* overexpression reduced high Pi-induced cell senescence, as shown by a lower level of senescence markers (p16 and p21) and SASP components (Il-6, Il-8, Cxcl1, Csf2, and Opg) (Fig. 5L and M), underscoring the potent role of NRF2 in reducing VSMC calcification and cellular senescence. Collectively, these results affirmed that *Nrf2* overexpression effectively counteracted VSMC calcification, diminishing cellular senescence.

### Transcriptional Profiles in Nrf2^SMCKO^ Mice Aorta

**Figure 6.**
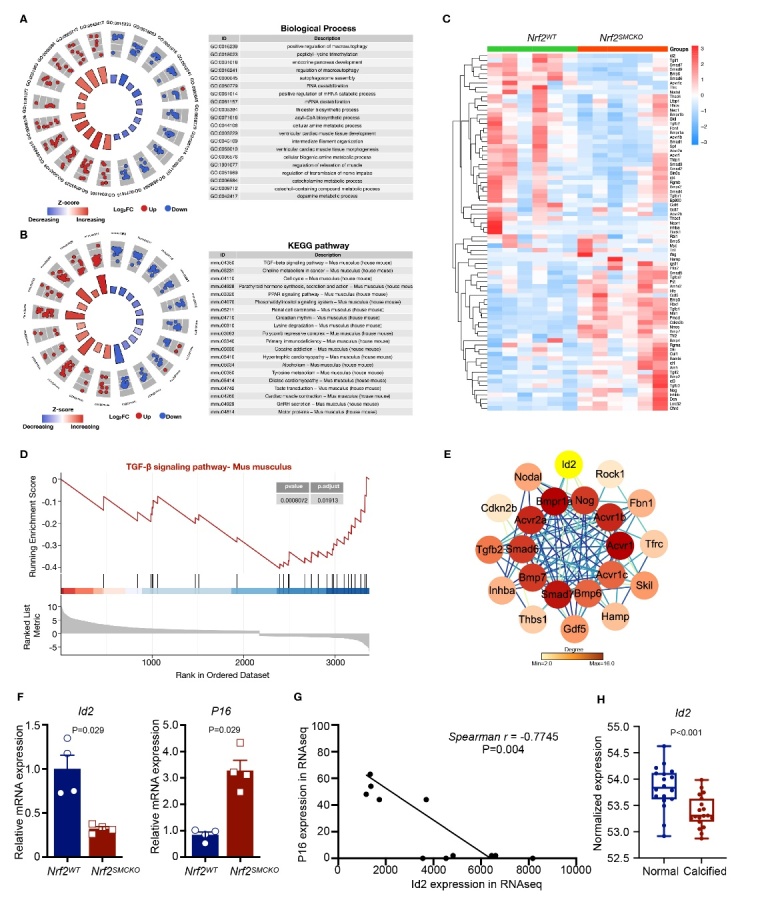
**Transcriptional Profiles in *Nrf2*^SMCKO^ Mice Aorta**. (**A-B**) The top 20 significantly enriched Gene Ontology Biological Processes (GO-BP) categories and KEGG pathways from differentially expressed genes (DEGs) between *Nrf2^SMCKO^* and *Nrf2^WT^* mouse aortas. (**C**) Heatmap illustrating DEGs enriched in the TGFβ signaling pathway. (**D**) Gene Set Enrichment Analysis (GSEA) of *Nrf2^SMCKO^* and *Nrf2^WT^* mouse aortas. (**E**) Protein-Protein Interactions (PPI) analysis of DEGs involved in the TGFβ signaling pathway. (**F**) Quantitative real-time PCR analysis of Id2 and p16 in *Nrf2^SMCKO^* and *Nrf2^WT^* mouse aortas (relative to β-actin, n=4 per group). (**G**) Correlation analysis between Id2 and p16 expression using RNA-seq data. (**H**) Scatterplots of Id2 expression in aortic samples from patients with calcified aortas (GSE12644 and GSE83453, n=18 and 19, respectively).

RNA sequencing (RNA-seq) was used to explore the downstream mechanisms of NRF2 in vascular protection. The dataset comprised 12 samples, including six aortas from *Nrf2^WT^* mice and six from *Nrf2^SMCKO^* mice. Of these, 3,477 differentially expressed genes (DEGs) were identified: 2,235 were upregulated and 1,242 were downregulated. Subsequent analysis using DAVID revealed the most significantly enriched Gene Ontology biological process (GO-BP) categories and KEGG pathways, as shown in Figure 6A and B. Notably, the TGF-β signaling pathway topped the list of enriched KEGG pathways. Further, Gene-set enrichment analysis (GSEA) illustrated that genes in the TGF-β signaling pathway were significantly overrepresented among DEGs in *Nrf2^SMCKO^* mice aortas compared to those in *Nrf2^WT^* mice, suggesting a potential regulatory role of NRF2 in this pathway (Fig. 6C and D). We then utilized the STRING database to analyze PPIs among DEGs in the TGF-β signaling pathway for visualization (Fig. 6E). I*d2* emerged as a core gene within the TGF-β signaling pathway and was markedly downregulated among the DEGs. In contrast, *P16* was significantly upregulated. Differential expression of both Id2 and p16 was confirmed in the aortic samples of *Nrf2^WT^* and *Nrf2^SMCKO^* mice, with decreased Id2 and increased p16 expression in the *Nrf2^SMCKO^* group (Fig. 6F). Additionally, a negative correlation between Id2 and p16 expression was observed in the RNA-seq data (Fig. 6G). We then analyzed the datasets (GSE12644 and GSE83453) obtained from aortas isolated from patients with stenosis for Id2 expression. Id2 was significantly downregulated in calcified aortas (Fig. 6H). These findings suggested a potential interaction between ID2 and P16 in the context of NRF2's protective function in the VC.

**Figure 7.**
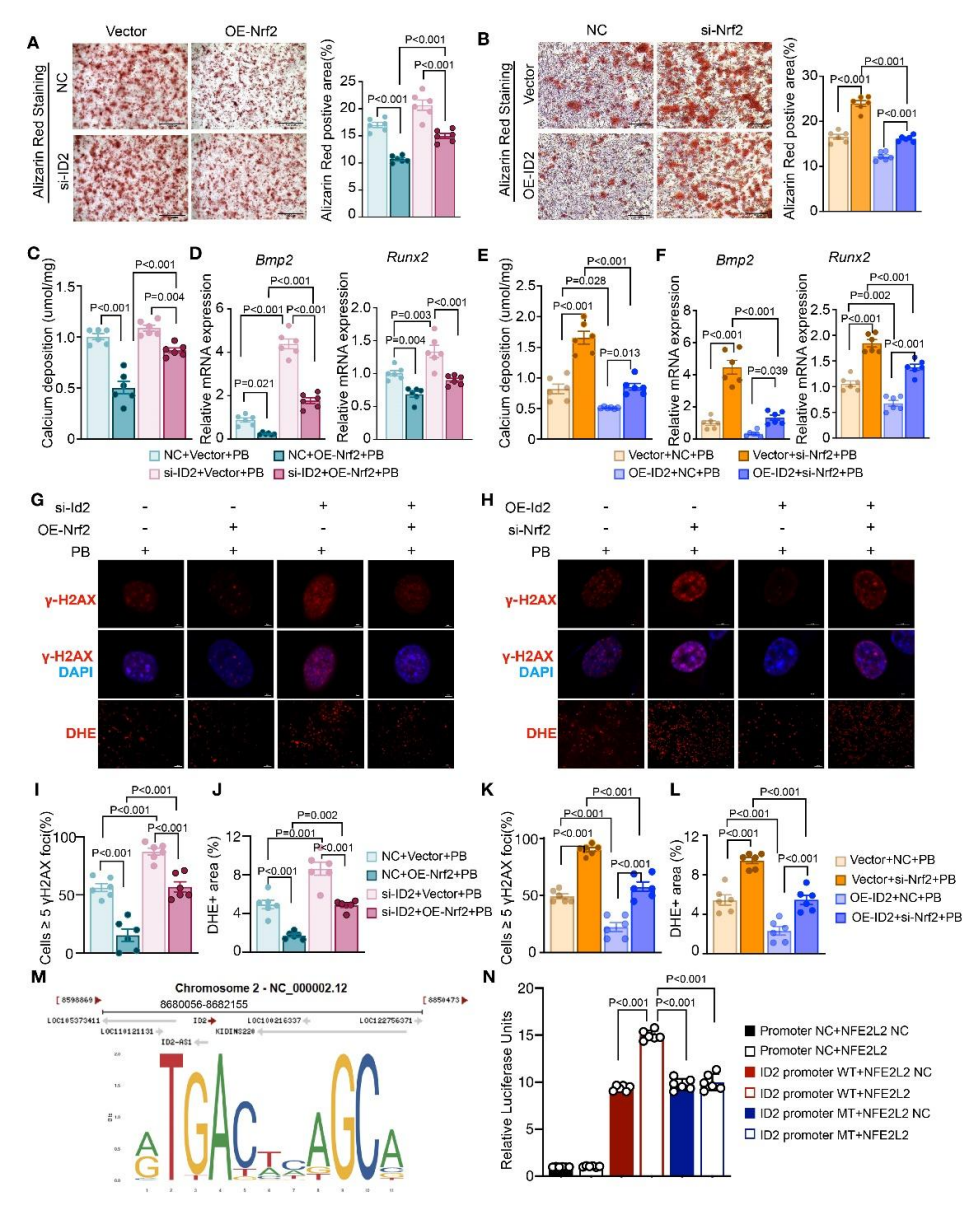
**ID2 Contributes to the Protective Effect of NRF2 in VC**. (**A**) Representative micrographs of Alizarin Red S staining (scale bars represent 1000μm) and quantification of the percentage of Alizarin Red S-positive area treated with Nrf2 lentivirus or control lentivirus, with Id2 siRNA or control siRNA, with High-Pi stimulation (n=6 per group). (**B**) Representative micrographs of Alizarin Red S staining (scale bars represent 100μm) and quantification of the percentage of Alizarin Red S-positive area treated with Id2 lentivirus or control lentivirus, with Nrf2 siRNA or control siRNA, with High-Pi stimulation (n=6 per group). (C, E) Quantitative analysis of calcium deposition under each treatment condition (n=6 per group). (D, F) Quantitative real-time PCR analysis of Bmp2 and Runx2 under each treatment condition (relative to β-actin, n=6 per group). (**G-L**) Representative micrographs of γ-H2AX (scale bars represent 5μm) and DHE staining (scale bars represent 500μm) under each condition, alongside quantification of the percentage of cells with 5 or more γ-H2AX foci and DHE-positive areas (n=6 per group). (**M**) Scanning the promoter of ID2 using the JASPAR website and predicted the potential binding site for NRF2. (**N**) A dual-luciferase reporting assay in PGL3-Basic vectors successfully constructed wild-type and mutant reporter plasmids in the ID2 promoter region (n=6 per group). ‘PB’ stands for ‘under high-phosphate condition’, ‘Vector’ stands for ‘OE-Control’, ‘NC’ stands for ‘si-Control’, and ‘Vector’ stands for ‘OE-Control’.

**Figure 8.**
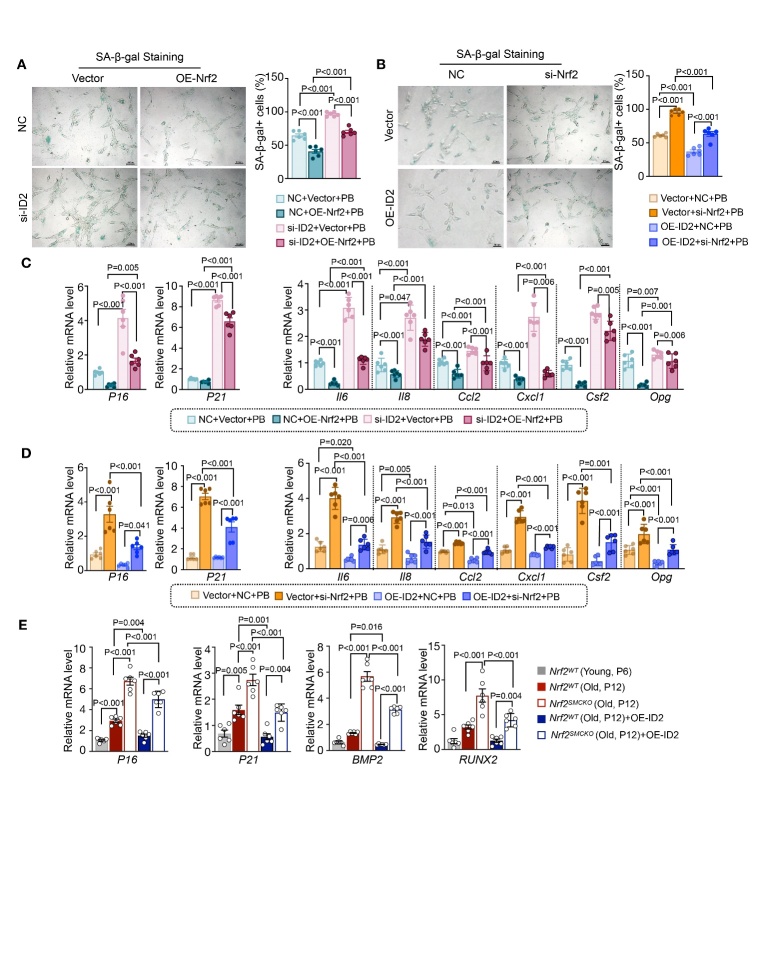
**NRF2-ID2 Axis Inhibited VC through Regulating VSMC Senescence**. (**A**) Representative micrographs of SA-β-gal staining (Scale bar represents 100μm) and quantification of the percentage of SA-β-gal positive cells area treated with Nrf2 lentivirus or control lentivirus, with Id2 siRNA or control siRNA, with High-Pi stimulation (n=6 per group). (**B**) Representative micrographs of SA-β-gal staining (Scale bar represents 100μm) and quantification of the percentage of SA-β-gal positive cells area treated with Id2 lentivirus or control lentivirus, with Nrf2 siRNA or control siRNA, with High-Pi stimulation (n=6 per group). (**C**) Quantitative real-time PCR analysis of cell senescence markers (p16 and p21) and SASP components (Il-6, Il-8, Ccl2, Cxcl1, Csf2, Opg) in MOVAS cells treated with Nrf2 lentivirus or control lentivirus, with Id2 siRNA or control siRNA, with High-Pi stimulation (relative to β-actin, n=6 per group). (**D**) Quantitative real-time PCR analysis of cell senescence markers (p16 and p21) and SASP components (Il-6, Il-8, Ccl2, Cxcl1, Csf2, Opg) in MOVAS cells treated with Id2 lentivirus or control lentivirus, with Nrf2 siRNA or control siRNA, with High-Pi stimulation (relative to β-actin, n=6 per group). (**E**) Quantitative real-time PCR analysis of p16, p21, Bmp2 and Runx2 in primary mouse VSMCs from *Nrf2^SMCKO^* or *Nrf2^WT^* mice, in passage 6 or passage 12 (relative to β-actin, n=6 per group). ‘PB’ stands for ‘under high-phosphate condition’, ‘Vector’ stands for ‘OE-Control’, ‘NC’ stands for ‘si-Control’, and ‘Vector’ stands for ‘OE-Control’.

### ID2 Contributes to the Protective Effect of NRF2 in VC

To investigate the potential link between the beneficial effects of ID2 and NRF2 in VC, we revisited both the Vitamin D-mediated VC mouse model and the high-Pi-induced VSMC calcification model. In *Nrf2^SMCKO^* mouse aortas, Id2 protein expression and nuclear translocation was significantly diminished compared to *Nrf2^WT^* counterparts. Vitamin D treatment exacerbated the reduction in ID2 protein expression and nuclear translocation in vivo (Supplementary Fig. 4A and B). In vitro, while high Pi diminished *Id2* expression in the mRNA level, *Nrf2* overexpression via LV-NRF2 notably elevated Id2 expression at both the mRNA and protein levels compared with LV-CON without high-phosphate intervention (Supplementary Fig. 4C and D). However, there was no significant change in ID2 expression level between the *Nrf2* overexpression group and the control group with high-phosphate intervention. This may suggest that during high-phosphate intervention, compared to the normal culture medium group, ID2 as a protective mechanism is gradually depleted, leading to no significant change in ID2 expression level. To further clarify the role of ID2 in Nrf2-mediated regulation of VSMC calcification, we silenced *Id2* in *Nrf2* overexpression VSMCs, and overexpressed *Id2* in *Nrf2* knockdown VSMCs. Successful downregulation and upregulation of *Id2* was verified at both the mRNA and protein levels (Supplementary Fig. 4E-H). Under high-Pi conditions, *Nrf2* overexpression significantly reduced calcium deposition and the Alizarin Red S-positive area (Figures 7A and 7C), along with a reduction in *Bmp2* and *Runx2* mRNA expression in VSMCs (Fig. 7D). Id2 knockdown compromised the Nrf2-mediated protective effects (Fig. 7A, C, and D). Nrf2 knockdown significantly accelerated calcium deposition and the Alizarin Red S-positive area (Fig. 7B and E), along with an increase in Bmp2 and Runx2 mRNA expression in VSMCs (Figure 7F). ID2 overexpression compromised the Nrf2-deficient induced pro-calcific effects (Fig. 7B, E and F). Further, under high-Pi conditions, *Id2* silencing undermined NRF2's efficacy in diminishing oxidative stress, as evidenced by DHE staining (Fig. 7G and J) and lessened the capacity of Nrf2 to mitigate DNA damage, as shown by γ-H2AX IF staining (Fig. 7G and I). While Id2 overexpression undermined Nrf2 deficient induced oxidative stress and DNA damage (Fig. 7H, K, and L). Moreover, we scanned the promoter of ID2 using the JASPAR website and predicted the potential binding site for NRF2 (Fig. 7M). A dual-luciferase reporting assay showed that PGL3-Basic vectors successfully constructed wild-type and mutant reporter plasmids in the ID2 promoter region. In HEK-293 cells, overexpression of transcription factor NRF2 enhanced the luciferase activity of PGL3-Basic-ID2-WT-luc reporter gene expression vector, indicating that transcription factor NRF2 promoted the transcription activity of the *ID2* gene promoter region (Fig. 7N). In combination with the above results of NRF2 regulation of ID2, we speculate that ID2, which is downstream of NRF2, contributes to the effect of NRF2 on VSMC calcification.

### NRF2-ID2 Axis Inhibited VC through Regulating VSMC Senescence

ID2 was found to be instrumental in Nrf2's suppression of VSMC senescence, indicated by SA-β-gal staining (Fig. 8A and B). RT-PCR analysis revealed that *Nrf2* overexpression led to a decrease in the expression of senescence markers (p16 and p21) and certain SASP components (Il-6, Ccl2, Cxcl1, Csf2, and Opg) under high-Pi conditions (Fig. 8C). *Id2* silencing increased the expression of these senescence markers and reversed the beneficial modulatory effects of NRF2 on p16, p21, and SASP components (Il-6, Il-8, Ccl2, Cxcl1, Csf2, and Opg) (Fig. 8C). While Nrf2 knockdown led to an increase in senescence markers (p16 and p21) and SASP components (Il-6, Il-8, Ccl2, Cxcl1, Csf2, and Opg) both with and without a high-Pi medium (Fig. 8D). *Id2* overexpression reduced the expression of these senescence markers and reversed the modulatory effects of NRF2 on p16, p21, and SASP components (Il-6, Il-8, Ccl2, Cxcl1, Csf2, and Opg) under high-Pi conditions (Fig. 8D).

To address this, we used mouse VSMCs replicative senescence models. Replicative senescence was induced by extended passages (passages 12) in mouse primary VSMCs, and cells in an early passage (passage 6) were used as young control cells. To determine whether senescent VSMCs accelerate calcification, we found that P16 and P21 levels were significantly increased in the senescent VSMCs group (Fig. 8E). Compared with the young control group, the expression of the osteoblast-like proteins BMP2 and RUNX2 was significantly increased in senescent VSMCs, suggesting that senescent VSMCs tend to undergo an osteoblastic transition and are more prone to calcification (Fig. 8E). To verify the role of the NRF2/ID2 axis in the osteoblastic transition of senescent cells, primary VSMCs isolated from *Nrf2*^SMCKO^ and *Nrf2*^WT^ mice, while *Id2* was overexpressed in senescent VSMCs. Knockdown of *Nrf2* significantly increased the expression of senescent markers (P16, P21) and osteoblastic markers (BMP2 and RUNX2) in senescent VSMCs (Fig. 8E). Furthermore, overexpression of *Id2* in the *Nrf2* knockdown group showed that ID2 could partially alleviate senescence and osteoblastic transition induced by *Nrf2* knockdown (Fig. 8E). These findings suggest a critical interaction between ID2 and NRF2 in VC, implying a significant role of VSMC senescence in this process.

## DISCUSSION

**Figure 9.**
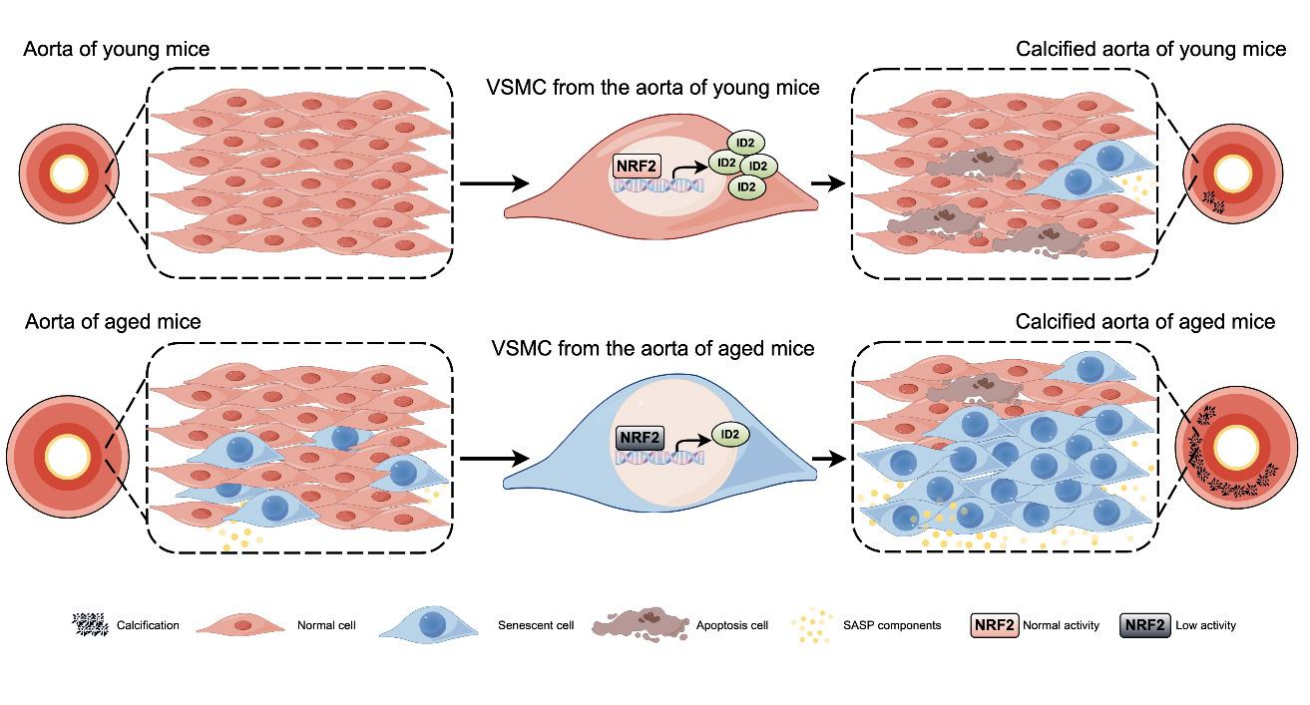
Graph Abstract of Our Findings.

Medial VC is a complex phenomenon commonly observed in individuals with chronic kidney disease, diabetes, and advancing age [30]. It contributes significantly to the pathophysiological progression of diseases by increasing arterial stiffness, consequently elevating the risk of cardiovascular morbidity and mortality [31]. OS is recognized as a pivotal regulator of this process, affecting VC through cell differentiation, apoptosis, senescence, inflammation, and extracellular matrix remodeling [11]. However, the precise mechanisms by which NRF2 influences VC progression and how NRF2 establishes a connection between aging and VC remain elusive. Our study revealed that aging accelerated medial VC in mice, coinciding with diminished NRF2 activity, increased VSMC senescence, and reduced apoptosis. Administering NRF2 activators effectively reduced calcification. Moreover, the specific knockout of *Nrf2* in VSMCs aggravated VC in various calcification models, whereas overexpression of *Nrf2* reduced calcium deposition and attenuated VSMC senescence. Through RNA-seq analysis of aortas from *Nrf2^SMCKO^* and control mice, we identified a novel downstream target of NRF2, ID2, which is indispensable for the protective effect of NRF2 in VC. NRF2 promotes the transcription activity of the *ID2* gene by binding it to its promoter region. In conclusion, our study sheds light on the accelerated calcification observed in the context of aging. The protective effects of Nrf2 on VSMC senescence and identification of ID2 as a downstream target contribute to our understanding of the complex mechanisms underlying VC (Fig. 9).

Various agents, including empagliflozin, metformin, rosmarinic acid, hydrogen sulfide, and dimethyl fumarate, have been shown to activate NRF2 effectively and counteract VC. These agents are used to mitigate high phosphate-induced calcification in VSMCs and CKD-induced aortic calcium deposition [15-19]. However, it is imperative to acknowledge that NRF2 activators lack specificity and may activate alternative pathways, such as the NF-κB signaling pathway, which has also been implicated in inhibiting VC [32, 33]. Molecules associated with NRF2, both upstream and downstream, that inhibit VC, have been identified. For example, glycosylation of KEAP1, which leads to NRF2 degradation, promotes VSMC calcification [21]. Conversely, activation of the NRF2/NQO1/HO-1 and NRF2/P62 pathways protects VSMCs against osteogenic transition and calcification [20, 22]. Nonetheless, the effect of NRF2 varies across different tissues and cell types. Its complex and sometimes ambiguous role in whole-body interventions complicates the identification of the site of its primary protective action. Therefore, creating tissue-specific knockout transgenic models is vital to precisely determine the role of NRF2 in vascular smooth muscle tissue, especially in the mechanisms of medial VC. In our study, we used a VSMC-specific *Nrf2* knockout mouse and demonstrated the protective effects of NRF2 in adenine- and Vitamin D-induced calcification models. Aortic rings and primary VSMCs from *Nrf2^SMCKO^* mice also showed increased VC under high-Pi conditions. These results provide robust evidence of the anti-calcification influence of NRF2 in VSMCs.

Imbalances between the oxidative and antioxidant systems are key characteristics of aging [34]. As a master regulator in the antioxidant response, NRF2 can activate numerous tissue-specific cytoprotective proteins throughout the lifespan of an organism [35, 36]. Nevertheless, aging impairs the inducible activity of NRF2 in the aorta of monkeys and rats, leading to increased ROS production [13, 14]. Our investigations revealed that aging diminished the expression and activity of NRF2 in the mouse aorta, thereby exacerbating OS. This underscores the crucial role of age-associated NRF2 dysfunction in the imbalance between oxidants and antioxidants. The arterial calcium level and the prevalence of cardiovascular disease increase with age [7]. However, most studies exploring the relationship between aging and VC are observational and lack experimental evidence for a comprehensive understanding. Using a mouse model with young and aged cohorts, our study demonstrated that aging accelerates VC, which is associated with increased OS, increased VSMC senescence, and reduced apoptosis. We also confirmed that NRF2 activators such as DMF and tBHQ can reverse VC in aged mouse aortas, positioning NRF2 as a key mediator in the nexus between aging and VC. Aging is associated with an accumulation of senescent cells resistant to apoptosis, contributing to medial calcification and increased expression of osteogenic markers [9, 37]. Silencing *Nrf2* has been found to accelerate the senescence of vascular cells [38, 39], with both senescent cell accumulation and NRF2 dysregulation implicated in medial VC pathogenesis [40]. Our study indicates that *Nrf2* silencing aggravates VSMC calcification with increased VSMC senescence and an osteogenic phenotype, whereas *Nrf2* overexpression inhibits VSMC calcification with decreased VSMC senescence and an osteogenic phenotype. A growing body of evidence suggests that activating NRF2 could provide protection against age-related VC by mitigating cellular senescence.

To explore the target genes of NRF2 in the VC, we performed transcriptome RNA-seq analysis of aortas from *Nrf2^SMCKO^* and *Nrf2^WT^* mice. The functional enrichment analyses of DEGs have indicated that the downregulated genes in *Nrf2^SMCKO^* mice aortas were primarily enriched in the TGFβ signaling pathway. The TGFβ superfamily, including TGF-βs, BMPs, and activins, is known to profoundly influence the initiation and progression of VC. An elevated TGFβ level, along with specific osteogenic BMPs, BMP2 and BMP4, are commonly seen in early medial calcified lesions, particularly in CKD-induced VC [41]. In contrast, BMP7 is a critical inhibitor of VC [42]. In our study, PPI analysis has identified ID2 as the core of DEGs enriched in the TGFβ signaling pathway. ID2, a DNA-binding protein inhibitor, plays a crucial role in cell proliferation and differentiation. It interacts with the retinoblastoma protein (pRb), neutralizing its growth-suppressing function and modulating cell differentiation. This interaction is particularly noteworthy given that ID2, but not ID1 or ID3, can counteract cell cycle arrest induced by p16, a known selective inhibitor of pRb and related kinases [43]. ID2 also promotes pro-survival and proliferative states in VSMCs in response to various stressors [44], underscoring its significance in cellular adaptation. Interestingly, ID2 responds to TGF-β/BMP signaling [45], influencing VSMC phenotype transitions from contractile to synthetic forms through the PI3K/AKT/ID2 pathway [46]. The BMP-SMAD-ID signaling cascade has been shown to counteract p16^INK4A^-mediated cell senescence, indicating a protective role against cellular aging [47]. Remarkably, ID2 has been identified as a pivotal factor in osteogenic differentiation. Prior studies have shown that ID2 suppresses osteogenesis in C2C12 cells by inhibiting the activation of core binding factor α-1(Cbfa1) [48]. Notably, an *Id2* knockout mouse model revealed that ID2 promotes cartilage formation by enhancing BMP signaling through a reduction in Smad7 expression [49]. Additionally, ID2 is known to curtail ROS production caused by glucose deprivation, alleviate mitochondrial damage, and decrease cell death caused by calcium overload [50]. In our study, we observed a decrease in *Id2* expression in *Nrf2^SMCKO^* mouse aortas. Conversely, overexpressing *Nrf2* led to the upregulation of ID2. Overexpression of ID2 alleviated NRF2 knockdown-induced VC and VSMC senescence while silencing ID2 negated the protective effect of NRF2 against VC and VSMC senescence. Moreover, a dual-luciferase reporting assay indicated that NRF2 promotes the transcription activity of the *ID2* gene by binding it to its promoter region. Interestingly, our study revealed decreased NRF2 expression in ID2-deficient VSMCs. Previous studies have demonstrated that ID2 activates NRF2 to protect retinal pigment epithelial cells from oxidative damage [51]. These findings highlight the intricate interplay between ID2 and NRF2 in the management of oxidative stress and cell senescence, underscoring their significant roles in vascular calcification.

In conclusion, our study reveals the critical role of the NRF2-ID2 axis in shielding VSMCs from calcification by counteracting VSMC senescence. The underlying molecular mechanisms present a significant potential for combating VC and open new avenues for understanding the relationship between aging and VC. However, given the intricate interplay between these pathways, further studies are required to fully understand the detailed molecular regulation of the NRF2-ID2 axis. Based on these detailed studies, we anticipate the development of novel therapeutic strategies against VC and age-related cardiovascular diseases.

## Supplementary Materials

The Supplementary data can be found online at: www.aginganddisease.org/EN/10.14336/AD.2024.0075.


