# Supplementary material for: The NRF2/ID2 Axis in Vascular Smooth Muscle Cells: Novel Insights into the Interplay between Vascular Calcification and Aging
Source: Aging Dis. 2024 May 20;16(2):1120–40. doi: 10.14336/AD.2024.0075 (PMC11964430; doi:10.14336/AD.2024.0075)
Supplement: Supplementary file 1 [file AD-16-2-1120-s.pdf]

SUPPLEMENTARY DATA

The NRF2/ID2 Axis in Vascular Smooth Muscle Cells: Novel Insights into the Interplay between Vascular Calcification and Aging

Mulin Xu, Xiuxian Wei, Jinli Wang, Yi Li, Yi Huang, Anying Cheng, Fan He, Le Zhang, Cuntai Zhang, Yu Liu

© 2024. Xu M et al. Published online at http://www.aginganddisease.org/EN/10.14336/AD.2024.0075

SUPPLEMENTARY DATA

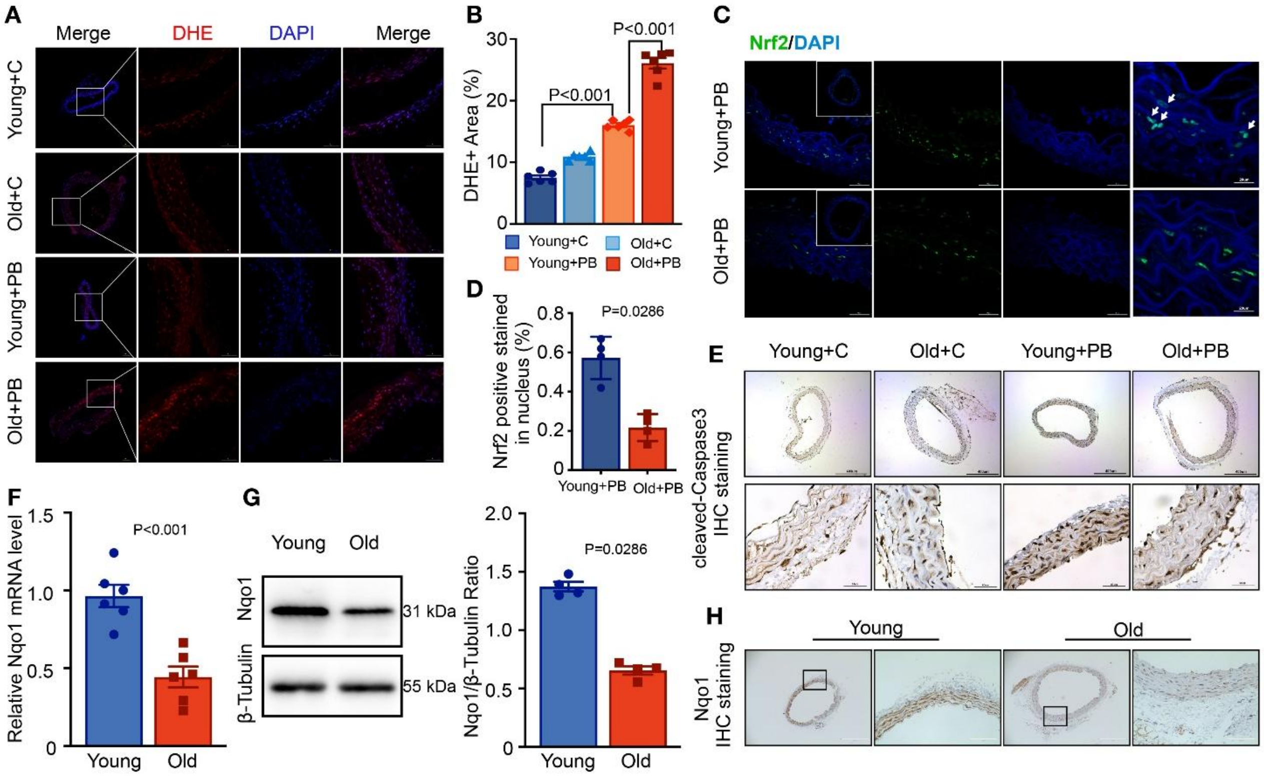

Supplementary Figure 1. Oxidative Stress and NRF2 Nuclear Translocation in Aged Mice Aortas. A, Representative micrographs of DHE staining (Scale bars represent 100μm and 50μm) in serial sections of aged and young mice aortas, both with and without 3.8 mmol/L High-Pi stimulation. **B,** Quantification of the percentage of DHE-positive areas in serial sections of aged and young mice aortas, both with and without 3.8 mmol/L High-Pi stimulation (n=6 per group). **C,** Immunofluorescence staining of NRF2 in serial sections of aged and young mice aortas under High-Pi stimulation (Scale bars represent 100μm, 50μm and 20μm). Arrows indicate areas of intranuclear NRF2 upregulation. **D,** Quantification of the percentage of Nrf2 positive-stained nucleus in serial sections of aged and young mice aortas with 3.8 mmol/L High-Pi stimulation (n=4 per group). **E,** Immunohistochemistry staining displaying cleaved-Caspase3 in serial sections of aged and young mice aortas under normal or high-phosphate (High-Pi) conditions (Scale bars represent 400μm and 50μm). **F,** Quantitative real-time PCR analysis of Nqo1 in aged and young mice aortas (relative to β-actin, n=6 per group). **G,** Western blot analysis of Nqo1 protein levels in aged and young mice aortas (relative to β-tubulin, n=4 per group). **H,** Immunohistochemistry staining displaying Nqo1 in serial sections of aged and young mice aortas under normal or high-phosphate (High-Pi) conditions (Scale bars represent 400μm and 100μm). 'C' stands for 'under normal condition', 'PB' stands for 'under high-phosphate condition'.

© 2024. Xu M et al. Published online at http://www.aginganddisease.org/EN/10.14336/AD.2024.0075

SUPPLEMENTARY DATA

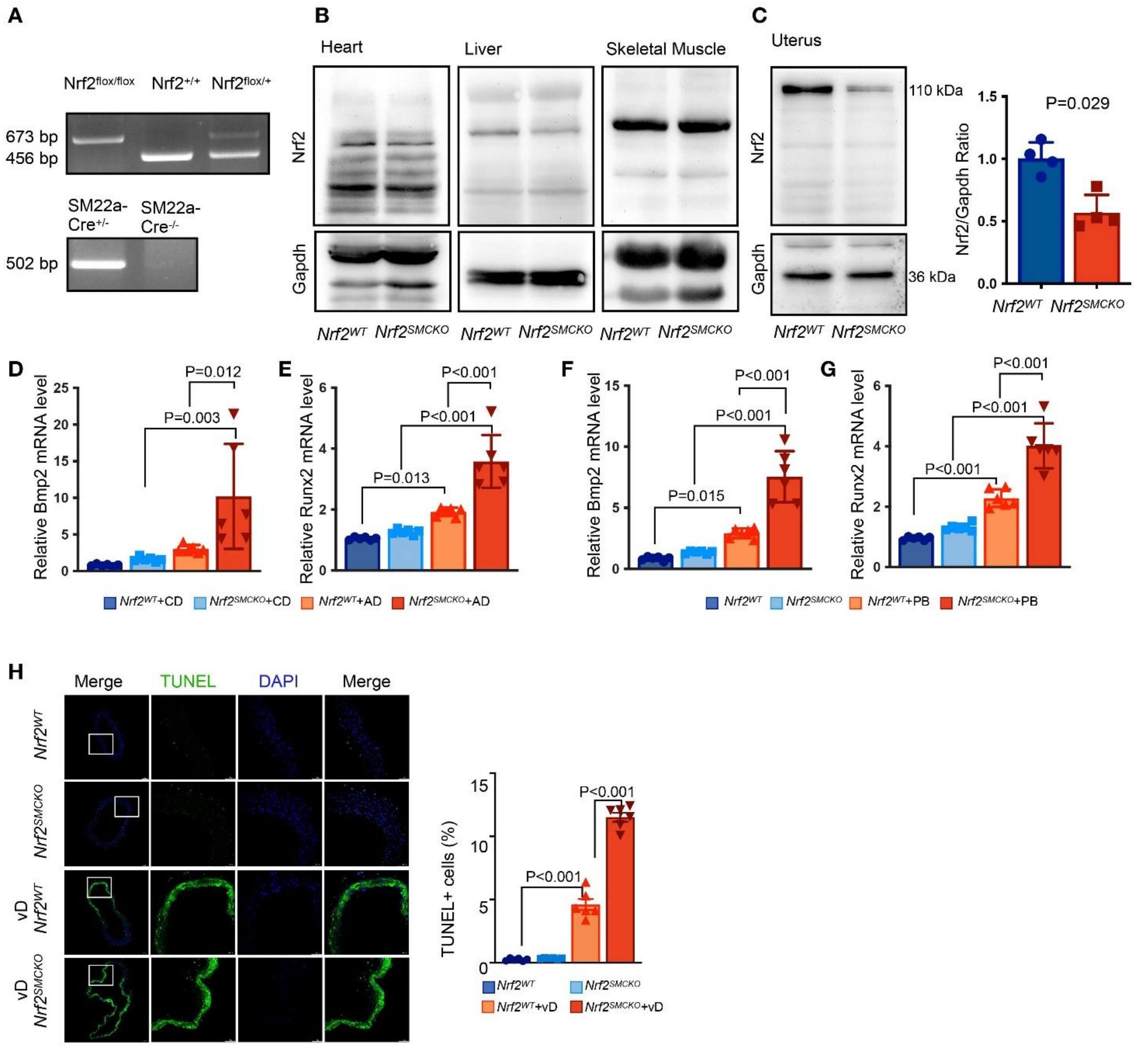

Supplementary Figure 2. PCR Genotyping and Western Blot Analysis of NRF2 for *$Nrf2^{SMCKO}$* Mice. A, PCR genotyping of genomic DNA from *$Nrf2^{SMCKO}$* (*$Tagln\text{-}Cre^{/+}$; $Nrf2^{flox/flox}$*) and *$Nrf2^{WT}$* (*$Tagln\text{-}Cre^{/-}$; $Nrf2^{flox/flox}$*) mice to confirm the specific knockout. **B,** Western blot analysis of NRF2 protein levels in the heart, liver, skeletal muscle of *$Nrf2^{SMCKO}$* and *$Nrf2^{WT}$* mice. **C,** Western blot analysis of NRF2 protein levels in the uterus of *$Nrf2^{SMCKO}$* and *$Nrf2^{WT}$* mice (relative to GAPDH, n=4 per group). **D,** Quantitative real-time PCR (relative to β-actin, n=6 per group) analyses assess the expression of Bmp2 in the aortas of *$Nrf2^{SMCKO}$* and *$Nrf2^{WT}$* mice with chow or adenine diet. **E,** Quantitative real-time PCR (relative to β-actin, n=6 per group) analyses assess the expression of Runx2 in the aortas of *$Nrf2^{SMCKO}$* and *$Nrf2^{WT}$* mice with chow or adenine diet. **F,** Quantitative real-time PCR (relative to β-actin, n=6 per group) analyses assess the expression of Bmp2 in the *$Nrf2^{SMCKO}$* and *$Nrf2^{WT}$* mice aortic rings cultured ex vivo with or without High-Pi treatment. **G,** Quantitative real-time PCR (relative to β-actin, n=6 per group) analyses assess the expression of Runx2 in the *$Nrf2^{SMCKO}$* and *$Nrf2^{WT}$* mice aortic rings cultured ex vivo with or without High-Pi treatment. **H,** Representative micrographs of TUNEL assay in serial sections of *$Nrf2^{SMCKO}$* and *$Nrf2^{WT}$* mice aortas following dextrose water or vitamin D treatment. Quantitative analysis of the percentage of TUNEL-positive cells in the aortas of *$Nrf2^{SMCKO}$* and *$Nrf2^{WT}$* mice treated with dextrose water or vitamin D (n=6 per group). Scale bars represent 100μm and 50μm. 'CD' stands for 'chow diet', 'AD' stands for 'adenine diet', 'PB' stands for 'under high-phosphate condition', and 'vD' stands for 'vitamin D treatment'.

© 2024. Xu M et al. Published online at http://www.aginganddisease.org/EN/10.14336/AD.2024.0075

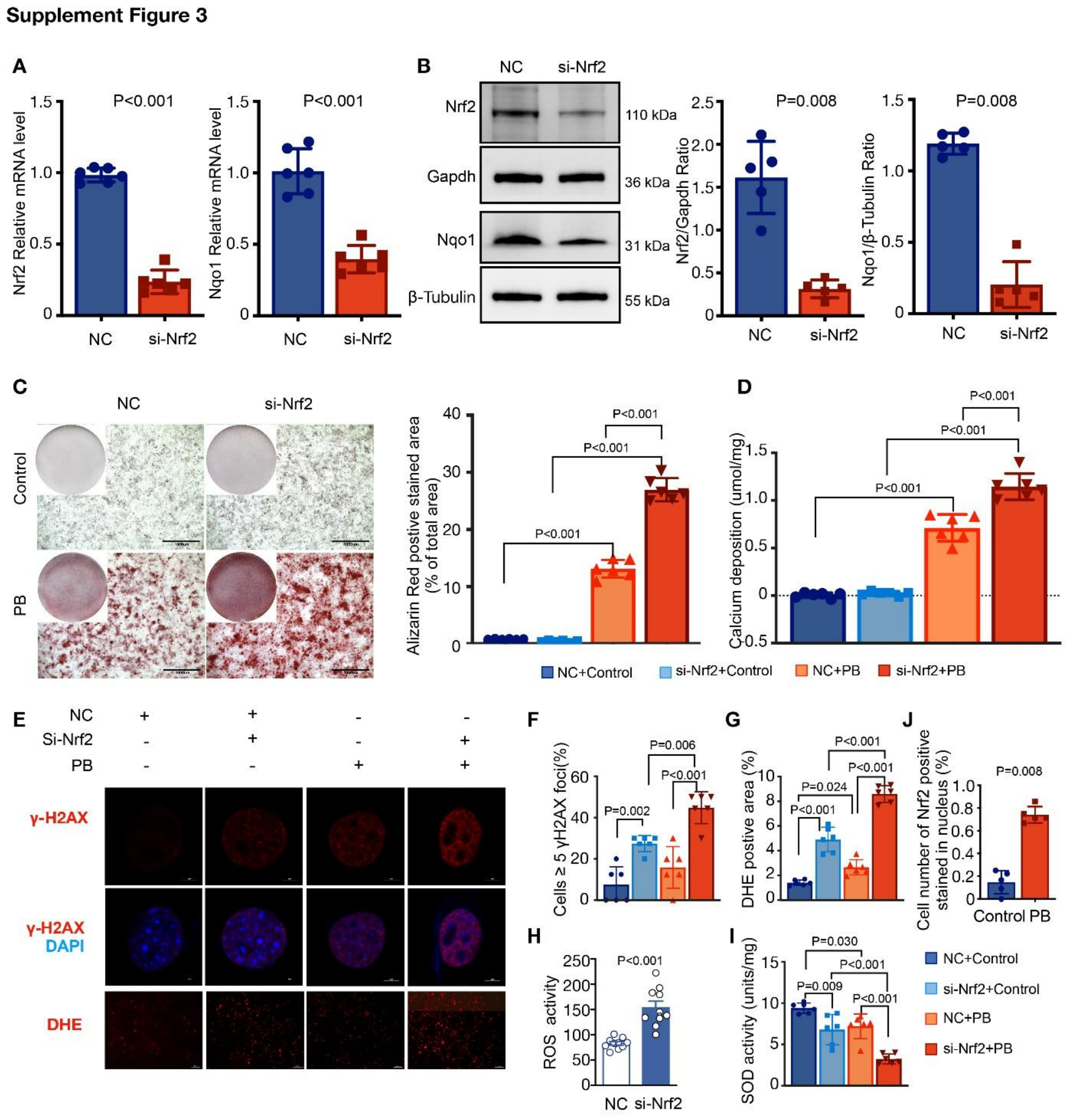

Supplementary Figure 3. ***Nrf2*** **Silencing Exacerbated VSMC Calcification. A-B,** Quantitative real-time PCR (relative to β-actin, n=6 per group) and Western blot (relative to GAPDH or β-tubulin, n=5 per group) analyses assess the expression of Nrf2 and Nqo1 in MOVAS cells treated with Nrf2 siRNA or control siRNA. **C,** Representative micrographs of Alizarin Red S staining, encompassing both whole well and microscopic views, along with quantification of the percentage of Alizarin Red S-positive area in MOVAS cells treated with Nrf2 siRNA or control siRNA, both with and without High-Pi stimulation (n=6 per group). **D,** Quantitative analysis of calcium deposition in MOVAS cells treated with Nrf2 siRNA or control siRNA, under both conditions (n=6 per group). **E,** Representative micrographs of γ-H_2AX (scale bars represent 5μm) and DHE staining (scale bars represent 500μm) in MOVAS cells treated with Nrf2 siRNA or control siRNA under both conditions. **F-G,** Quantitative analysis of cells with 5 or more γ-H_2AX foci and DHE-positive areas in MOVAS cells treated with Nrf2 siRNA or control siRNA, with or without High-Pi stimulation (n=6 per group). **H,** Detection of intracellular ROS (reactive oxygen species) levels using DCFH-DA in MOVAS cells treated with Nrf2 siRNA or control siRNA, with or without High-Pi stimulation (n=12 per group). Scale bar represents 20μm. **I,** Measurement of SOD (superoxide dismutase) activity in MOVAS cells treated with Nrf2 siRNA or control siRNA under both conditions (n=6 per group). **J,** Quantification of the percentage of Nrf2 positive-stained nucleus with or without High-Pi stimulation (n=5 per group). 'NC' stands for 'si-Control', 'PB' stands for 'under high-phosphate condition'.

© 2024. Xu M et al. Published online at http://www.aginganddisease.org/EN/10.14336/AD.2024.0075

SUPPLEMENTARY DATA

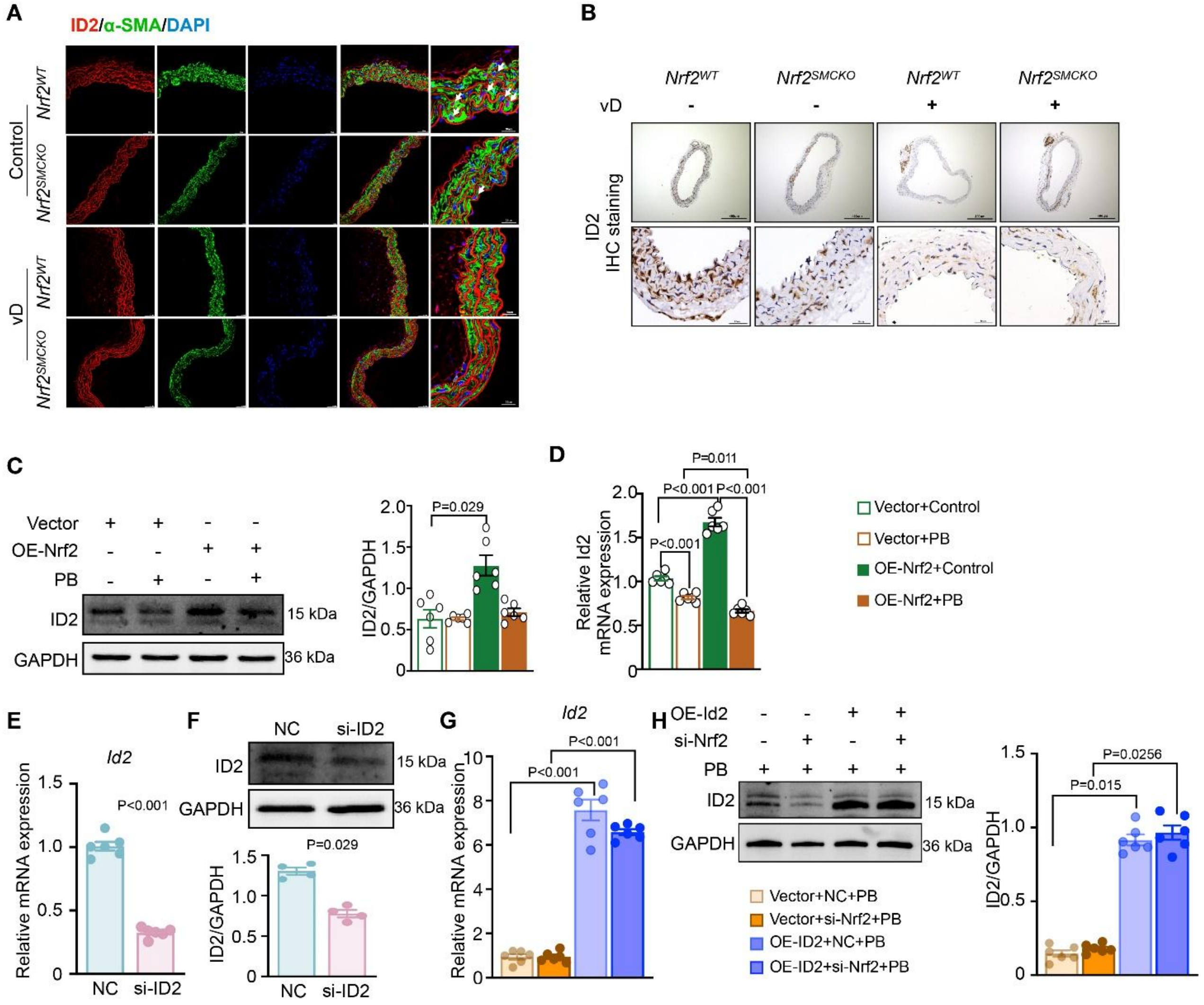

Supplementary Figure 4. ***Id2*** **Expression in** ***Nrf2*****^{SMCKO} Mouse Aortas and MOVAS. A,** Immunofluorescence staining for Id2 (red) and α-SMA (green) in serial sections of *Nrf2^{SMCKO}* and *Nrf2^{WT}* mouse aortas following injection with dextrose water or vitamin D (scale bars represent 50μm and 20μm). Arrows indicate areas of intranuclear Id2 upregulation. **B,** Immunohistochemistry staining displaying Id2 in serial sections of *Nrf2^{SMCKO}* and *Nrf2^{WT}* mouse aortas following injection with dextrose water or vitamin D (Scale bars represent 400μm and 50μm). **C-D,** Quantitative real-time PCR (relative to β-actin, n=6 per group) and Western blot (relative to GAPDH, n=6 per group) analyses of Id2 in MOVAS cells treated with NRF2 lentivirus or control lentivirus, with or without high-Pi stimulation. **E-F,** Quantitative real-time PCR (relative to β-actin, n=6 per group) and Western blot (relative to GAPDH, n=4 per group) analyses of Id2 expression in MOVAS cells treated with Id2 siRNA or control siRNA (n=5 per group). **G-H,** Quantitative real-time PCR (relative to β-actin, n=6 per group) and Western blot (relative to GAPDH, n=6 per group) analyses of Id2 expression in MOVAS cells treated with Id2 lentivirus or control lentivirus, with or without High-Pi stimulation. Scale bars represent 50μm and 20μm. 'vD' stands for 'vitamin D treatment', 'PB' stands for 'under high-phosphate condition', and 'Vector' stands for 'OE-Control'.

© 2024. Xu M et al. Published online at http://www.aginganddisease.org/EN/10.14336/AD.2024.0075

SUPPLEMENTARY DATA

Supplementary Table 1. Primers Applied in Real-time PCR.

Gene name	Species	Forward (5'-3')	Reverse (5'-3')
Nrf2	Mouse	AGTCGCTTGCCCTGGATA	GCTGCTTGTTTTCGGTATT
Nqo1	Mouse	CTTTAGGGTCGTCTTGGC	CAATCAGGGCTCTTCTCG
Hmox1	Mouse	GATAGAGCGCAACAAGCAGAA	CAGTGAGGCCCATACCAGAAG
Bmp2	Mouse	GGGACCCGCTGTCTTCTAGT	TCAACTCAAATTCGCTGAGGAC
Runx2	Mouse	AACGATCTGAGATTTGTGGGC	CCTGCGTGGGATTTCTTGGTT
p16	Mouse	CGCAGGTTCTTGGTCACTGT	TGTTCACGAAAGCCAGAGCG
p21	Mouse	CCTGGTGATGTCCGACCTG	CCATGAGCGCATCGCAATC
Il-6	Mouse	CCAAGAGGTGAGTGCTTCCC	CTGTTGTTCAGACTCTCTCCCT
Il-8	Mouse	CAAGGCTGGTCCATGCTCC	TGCTATCACTTCCTTTCTGTTGC
Ccl2	Mouse	TTAAAAACCTGGATCGGAACCAA	GCATTAGCTTCAGATTTACGGGT
Cxcl1	Mouse	CTGGGATTCACCTCAAGAACATC	CAGGGTCAAGGCAAGCCTC
Csf2	Mouse	GGCCTTGGAAGCATGTAGAGG	GGAGAACTCGTTAGAGACGACTT
Opg	Mouse	ACCCAGAAACTGGTCATCAGC	CTGCAATACACACACTCATCACT
Id2	Mouse	TACAACATGAACGACTGCTACT	ATTTCCATCTTGGTCACCTTCT
Tgfb1	Mouse	GTGCGGCAGCTGTACATTGACTTT	GGCTTGCGACCCACGTAGTAGAC
Actb	Mouse	TCAAGATCATTGCTCCTCCTGAG	ACATCTGCTGGAAGGTGGACA

Supplementary Table 2. Statistical information.

Figure	Panel	Number	Shapiro-Wilk normality test	Statistical test
Figure 1	1B Quantification of calcium deposition	6;10;6;10	passed	Parametric two-way ANOVA with post Tukey's multiple comparisons test
	1E Quantification of TUNEL staining	6;6;6;6	passed	Parametric two-way ANOVA with post Tukey's multiple comparisons test
	1I Western Blot of cleaved-Caspase3	4;4;4;4	/	Non-parametric Kruskal-Wallis test with post Dunn's multiple comparisons test
	1I Western Blot of Caspase3	4;4;4;4	/	Non-parametric Kruskal-Wallis test with post Dunn's multiple comparisons test
	1J mRNA of Nrf2	6;6	passed	unpaired Student’s t-test
	1K Western Blot of Nrf2	4;4	/	Non-parametric Mann Whitney test
	1M Quantification of calcium deposition with DMF	6;6;6;6	passed	Parametric two-way ANOVA with post Tukey's multiple comparisons test
	1N Quantification of calcium deposition with tBHQ	6;6;6;6	passed	Parametric two-way ANOVA with post Tukey's multiple comparisons test
Figure 2	2B Western Blot of Nrf2	4;4	/	Non-parametric Mann Whitney test
	2E Quantification of calcium deposition with chow or adenine diet	8;8;8;8	passed	Parametric two-way ANOVA with post Tukey's multiple comparisons test
	2G Quantification of calcium deposition with or without High-Pi stimulation.	6;6;6;6	passed	Parametric two-way ANOVA with post Tukey's multiple comparisons test
Figure 3	3C Quantification of calcium deposition following dextrose water or vitamin D injection	8;8;8;8	passed	Parametric two-way ANOVA with post Tukey's multiple comparisons test
	3E mRNA of Nrf2	6;6;6;6	passed	Parametric two-way ANOVA with post Tukey's multiple comparisons test
	3E mRNA of Nqo1	6;6;6;6	passed	Parametric two-way ANOVA with post Tukey's multiple comparisons test
	3E mRNA of Bmp2	6;6;6;6	passed	Parametric two-way ANOVA with post Tukey's multiple comparisons test

© 2024. Xu M et al. Published online at http://www.aginganddisease.org/EN/10.14336/AD.2024.0075

SUPPLEMENTARY DATA

	3E mRNA of Runx2	6;6;6;6	passed	Parametric two-way ANOVA with post Tukey's multiple comparisons test
	3H Quantification of DHE staining	6;6;6;6	passed	Parametric two-way ANOVA with post Tukey's multiple comparisons test
	3I mRNA of P16	6;6;6;6	passed	Parametric two-way ANOVA with post Tukey's multiple comparisons test
	3I mRNA of P21	6;6;6;6	passed	Parametric two-way ANOVA with post Tukey's multiple comparisons test
	3J mRNA of IL-6	6;6;6;6	passed	Parametric two-way ANOVA with post Tukey's multiple comparisons test
	3J mRNA of IL-8	6;6;6;6	passed	Parametric two-way ANOVA with post Tukey's multiple comparisons test
	3J mRNA of Ccl2	6;6;6;6	passed	Parametric two-way ANOVA with post Tukey's multiple comparisons test
	3J mRNA of Cxcl1	6;6;6;6	passed	Parametric two-way ANOVA with post Tukey's multiple comparisons test
	3J mRNA of Csf2	6;6;6;6	passed	Parametric two-way ANOVA with post Tukey's multiple comparisons test
	3J mRNA of Opg	6;6;6;6	passed	Parametric two-way ANOVA with post Tukey's multiple comparisons test
Figure 4	4A mRNA of Nrf2	6;6	passed	unpaired Student's t-test
	4A mRNA of Nqo1	6;6	passed	unpaired Student's t-test
	4B Western Blot of Nrf2	4;4	/	Non-parametric Mann Whitney test
	4B Western Blot of Nqo1	4;4	/	Non-parametric Mann Whitney test
	4C Quantification of Alizarin Red S staining	6;6;6;6	passed	Parametric two-way ANOVA with post Tukey's multiple comparisons test
	4D Quantification of calcium deposition	6;6;6;6	passed	Parametric two-way ANOVA with post Tukey's multiple comparisons test
Figure 5	5A mRNA of Nrf2	6;6;6;6	passed	Parametric two-way ANOVA with post Tukey's multiple comparisons test
	5A mRNA of Nqo1	6;6;6;6	passed	Parametric two-way ANOVA with post Tukey's multiple comparisons test
	5B Western Blot of Nrf2	4;4;4;4	/	Non-parametric Kruskal-Wallis test with post Dunn's multiple comparisons test
	5B Western Blot of Nqo1	4;4;4;4	/	Non-parametric Kruskal-Wallis test with post Dunn's multiple comparisons test
	5D Quantification of Alizarin Red S staining	6;6;6;6	passed	Parametric two-way ANOVA with post Tukey's multiple comparisons test
	5E Quantification of calcium deposition	6;6;6;6	passed	Parametric two-way ANOVA with post Tukey's multiple comparisons test
	5G Quantification of γ-H2AX staining	6;6;6;6	passed	Parametric two-way ANOVA with post Tukey's multiple comparisons test
	5H Quantification of DHE staining	6;6;6;6	passed	Parametric two-way ANOVA with post Tukey's multiple comparisons test
	5I Quantification of SOD activity	6;6;6;6	passed	Parametric two-way ANOVA with post Tukey's multiple comparisons test
	5J Quantification of ROS levels	12;12	passed	unpaired Student's t-test
	5K Quantification of SA-β-gal staining	6;6;6;6	passed	Parametric two-way ANOVA with post Tukey's multiple comparisons test
	5L mRNA of P16	6;6;6;6	passed	Parametric two-way ANOVA with post Tukey's multiple comparisons test
	5L mRNA of P21	6;6;6;6	passed	Parametric two-way ANOVA with post Tukey's multiple comparisons test
	5M mRNA of IL-6	6;6;6;6	passed	Parametric two-way ANOVA with post Tukey's multiple comparisons test
	5M mRNA of IL-8	6;6;6;6	passed	Parametric two-way ANOVA with post Tukey's multiple comparisons test
	5M mRNA of Ccl2	6;6;6;6	passed	Parametric two-way ANOVA with post Tukey's multiple comparisons test
	5M mRNA of Cxcl1	6;6;6;6	passed	Parametric two-way ANOVA with post Tukey's multiple comparisons test
	5M mRNA of Csf2	6;6;6;6	passed	Parametric two-way ANOVA with post Tukey's multiple comparisons test
	5M mRNA of Opg	6;6;6;6	passed	Parametric two-way ANOVA with post Tukey's multiple comparisons test
Figure 6	6F mRNA of Id2	4;4	/	Non-parametric Mann Whitney test

© 2024. Xu M et al. Published online at http://www.aginganddisease.org/EN/10.14336/AD.2024.0075

	6F mRNA of P16	4;4	/	Non-parametric Mann Whitney test
	6G Correlation analysis between Id2 and p16 expression	12;12	No	Spearman Correlation Analysis
	6H Id2 expression in aortic samples from patients with calcified aortas	18;19	passed	unpaired Student's t-test
Figure 7	7A Quantification of Alizarin Red S staining with Nrf2 lentivirus or control lentivirus, with Id2 siRNA or control siRNA	6;6;6;6	passed	Parametric two-way ANOVA with post Tukey's multiple comparisons test
	7B Quantification of Alizarin Red S staining with Id2 lentivirus or control lentivirus, with Nrf2 siRNA or control siRNA	6;6;6;6	passed	Parametric two-way ANOVA with post Tukey's multiple comparisons test
	7C Quantification of calcium deposition with Nrf2 lentivirus or control lentivirus, with Id2 siRNA or control siRNA	6;6;6;6	passed	Parametric two-way ANOVA with post Tukey's multiple comparisons test
	7D mRNA of Bmp2 with Nrf2 lentivirus or control lentivirus, with Id2 siRNA or control siRNA	6;6;6;6	passed	Parametric two-way ANOVA with post Tukey's multiple comparisons test
	7D mRNA of Runx2 with Nrf2 lentivirus or control lentivirus, with Id2 siRNA or control siRNA	6;6;6;6	passed	Parametric two-way ANOVA with post Tukey's multiple comparisons test
	7E Quantification of calcium deposition with Id2 lentivirus or control lentivirus, with Nrf2 siRNA or control siRNA	6;6;6;6	passed	Parametric two-way ANOVA with post Tukey's multiple comparisons test
	7F mRNA of Bmp2 with Id2 lentivirus or control lentivirus, with Nrf2 siRNA or control siRNA	6;6;6;6	passed	Parametric two-way ANOVA with post Tukey's multiple comparisons test
	7F mRNA of Runx2 with Id2 lentivirus or control lentivirus, with Nrf2 siRNA or control siRNA	6;6;6;6	passed	Parametric two-way ANOVA with post Tukey's multiple comparisons test
	7I Quantification of γ-H2AX staining	6;6;6;6	passed	Parametric two-way ANOVA with post Tukey's multiple comparisons test
	7J Quantification of DHE staining	6;6;6;6	passed	Parametric two-way ANOVA with post Tukey's multiple comparisons test
	7K Quantification of γ-H2AX staining	6;6;6;6	passed	Parametric two-way ANOVA with post Tukey's multiple comparisons test
	7L Quantification of DHE staining	6;6;6;6	passed	Parametric two-way ANOVA with post Tukey's multiple comparisons test
	7N Dual-luciferase reporting assay	6;6;6;6;6;6	passed	Parametric two-way ANOVA with post Tukey's multiple comparisons test
Figure 8	8A Quantification of SA-β-gal staining with Nrf2 lentivirus or control lentivirus, with Id2 siRNA or control siRNA	6;6;6;6	passed	Parametric two-way ANOVA with post Tukey's multiple comparisons test
	8B Quantification of SA-β-gal staining with Id2 lentivirus or control lentivirus, with Nrf2 siRNA or control siRNA	6;6;6;6	passed	Parametric two-way ANOVA with post Tukey's multiple comparisons test
	8C mRNA of P16 with Nrf2 lentivirus or control lentivirus, with Id2 siRNA or control siRNA	6;6;6;6	passed	Parametric two-way ANOVA with post Tukey's multiple comparisons test
	8C mRNA of P21 with Nrf2 lentivirus or control lentivirus, with Id2 siRNA or control siRNA	6;6;6;6	passed	Parametric two-way ANOVA with post Tukey's multiple comparisons test
	8C mRNA of IL-6 with Nrf2 lentivirus or control lentivirus, with Id2 siRNA or control siRNA	6;6;6;6	passed	Parametric two-way ANOVA with post Tukey's multiple comparisons test
	8C mRNA of IL-8 with Nrf2 lentivirus or control lentivirus, with Id2 siRNA or control siRNA	6;6;6;6	passed	Parametric two-way ANOVA with post Tukey's multiple comparisons test
	8C mRNA of Ccl2 with Nrf2 lentivirus or control lentivirus, with Id2 siRNA or control siRNA	6;6;6;6	passed	Parametric two-way ANOVA with post Tukey's multiple comparisons test
	8C mRNA of Cxcl1 with Nrf2 lentivirus or control lentivirus, with Id2 siRNA or control siRNA	6;6;6;6	passed	Parametric two-way ANOVA with post Tukey's multiple comparisons test
	8C mRNA of Csf2 with Nrf2 lentivirus or control lentivirus, with Id2 siRNA or control siRNA	6;6;6;6	passed	Parametric two-way ANOVA with post Tukey's multiple comparisons test
	8C mRNA of Opg with Nrf2 lentivirus or control lentivirus, with Id2 siRNA or control siRNA	6;6;6;6	passed	Parametric two-way ANOVA with post Tukey's multiple comparisons test
	8D mRNA of P16 with Id2 lentivirus or control lentivirus, with Nrf2 siRNA or control siRNA	6;6;6;6	passed	Parametric two-way ANOVA with post Tukey's multiple comparisons test
	8D mRNA of P21 with Id2 lentivirus or control lentivirus, with Nrf2 siRNA or control siRNA	6;6;6;6	passed	Parametric two-way ANOVA with post Tukey's multiple comparisons test
	8D mRNA of IL-6 with Id2 lentivirus or control lentivirus, with Nrf2 siRNA or control siRNA	6;6;6;6	passed	Parametric two-way ANOVA with post Tukey's multiple comparisons test
	8D mRNA of IL-8 with Id2 lentivirus or control lentivirus, with Nrf2 siRNA or control siRNA	6;6;6;6	passed	Parametric two-way ANOVA with post Tukey's multiple comparisons test
	8D mRNA of Ccl2 with Id2 lentivirus or control lentivirus, with Nrf2 siRNA or control siRNA	6;6;6;6	passed	Parametric two-way ANOVA with post Tukey's multiple comparisons test
	8D mRNA of Cxcl1 with Id2 lentivirus or control lentivirus, with Nrf2 siRNA or control siRNA	6;6;6;6	passed	Parametric two-way ANOVA with post Tukey's multiple comparisons test

	8D mRNA of Csf2 with Id2 lentivirus or control lentivirus, with Nrf2 siRNA or control siRNA	6;6;6;6	passed	Parametric two-way ANOVA with post Tukey's multiple comparisons test
	8D mRNA of Opg with Id2 lentivirus or control lentivirus, with Nrf2 siRNA or control siRNA	6;6;6;6	passed	Parametric two-way ANOVA with post Tukey's multiple comparisons test
	8E mRNA of P16	6;6;6;6	passed	Parametric two-way ANOVA with post Tukey's multiple comparisons test
	8E mRNA of P21	6;6;6;6	passed	Parametric two-way ANOVA with post Tukey's multiple comparisons test
	8E mRNA of Bmp2	6;6;6;6	passed	Parametric two-way ANOVA with post Tukey's multiple comparisons test
	8E mRNA of Runx2	6;6;6;6	passed	Parametric two-way ANOVA with post Tukey's multiple comparisons test
Figure S1	S1B Quantification of DHE staining	6;6;6;6	passed	Parametric two-way ANOVA with post Tukey's multiple comparisons test
	S1D Quantification of NRF2 nuclear staining	4;4	/	Non-parametric Mann Whitney test
	S1F mRNA of Nqo1	6;6	passed	unpaired Student's t-test
	S1G Western Blot of Nqo1	4;4	/	Non-parametric Mann Whitney test
Figure S2	S2C Western Blot of Nrf2 in the uterus	4;4	/	Non-parametric Mann Whitney test
	S2D mRNA of Bmp2 with chow or adenine diet	6;6;6;6	passed	Parametric two-way ANOVA with post Tukey's multiple comparisons test
	S2E mRNA of Runx2 with chow or adenine diet	6;6;6;6	passed	Parametric two-way ANOVA with post Tukey's multiple comparisons test
	S2F mRNA of Bmp2 with or without High-Pi treatment	6;6;6;6	passed	Parametric two-way ANOVA with post Tukey's multiple comparisons test
	S2G mRNA of Runx2 with or without High-Pi treatment	6;6;6;6	passed	Parametric two-way ANOVA with post Tukey's multiple comparisons test
	S2H Quantification of TUNEL staining	6;6;6;6	passed	Parametric two-way ANOVA with post Tukey's multiple comparisons test
Figure S3	S3A mRNA of Nrf2	6;6	passed	unpaired Student's t-test
	S3A mRNA of Nqo1	6;6	passed	unpaired Student's t-test
	S3B Western Blot of Nrf2	5;5	/	Non-parametric Mann Whitney test
	S3B Western Blot of Nqo1	5;5	/	Non-parametric Mann Whitney test
	S3C Quantification of Alizarin Red S staining	6;6;6;6	passed	Parametric two-way ANOVA with post Tukey's multiple comparisons test
	S3D Quantification of calcium deposition	6;6;6;6	passed	Parametric two-way ANOVA with post Tukey's multiple comparisons test
	S3F Quantification of γ-H2AX staining	6;6;6;6	passed	Parametric two-way ANOVA with post Tukey's multiple comparisons test
	S3G Quantification of DHE staining	6;6;6;6	passed	Parametric two-way ANOVA with post Tukey's multiple comparisons test
	S3H Quantification of ROS levels	12;12	passed	unpaired Student's t-test
	S3J Quantification of SOD activity	6;6;6;6	passed	Parametric two-way ANOVA with post Tukey's multiple comparisons test
	S3J Quantification of Nrf2 positive stained in nucleus	5;5	/	Non-parametric Mann Whitney test
Figure S4	S4C Western Blot of Id2	6;6;6;6	passed	Parametric two-way ANOVA with post Tukey's multiple comparisons test
	S4D mRNA of Id2	6;6;6;6	passed	Parametric two-way ANOVA with post Tukey's multiple comparisons test
	S4E mRNA of Id2	6;6	passed	unpaired Student's t-test
	S4F Western Blot of Id2	4;4	/	Non-parametric Mann Whitney test
	S4G mRNA of Id2	6;6;6;6	passed	Parametric two-way ANOVA with post Tukey's multiple comparisons test
	S4H Western Blot of Id2	6;6;6;6	passed	Parametric two-way ANOVA with post Tukey's multiple comparisons test

© 2024. Xu M et al. Published online at http://www.aginganddisease.org/EN/10.14336/AD.2024.0075
